# Dopamine alters functional gradients in Parkinson’s disease

**DOI:** 10.1162/imag_a_00564

**Published:** 2025-05-06

**Authors:** Isabella F. Orlando, Joshua B. Tan, Natasha L. Taylor, Vicente Medel, Gabriel Wainstein, Simon J.G. Lewis, James M. Shine, Claire O’Callaghan

**Affiliations:** Brain and Mind Centre and School of Medical Sciences, Faculty of Medicine and Health, University of Sydney, Sydney, Australia; Latin American Brain Health Institute (BrainLat), Universidad Adolfo Ibáñez, Santiago, Chile; Macquarie University Centre for Parkinson’s Disease Research and Macquarie Medical School, Faculty of Medicine, Health and Human Sciences, Macquarie University, Sydney, New South Wales, Australia; Centre for Complex Systems, School of Physics, University of Sydney, Sydney, Australia

**Keywords:** Parkinson’s disease, dopamine, resting-state fMRI, functional gradients, receptor expression

## Abstract

Neuromodulators regulate large-scale brain network topology to support adaptive behaviour. Disease models offer a unique window into how neuromodulatory systems impact large-scale brain network organisation. Here, we take advantage of Parkinson’s disease—with its profound dopaminergic loss and pro-dopaminergic treatment strategies—to inform how dopamine may influence large-scale brain organisation. In 27 people with Parkinson’s disease, resting-state scans were obtained on their regular dopamine medication and following overnight withdrawal of medication. Nineteen matched controls provided normative data. Gradients of brain organisation were examined using dimensionality reduction techniques. For single gradients, when individuals were on their dopamine medication, we observed a shift in higher-order networks towards somatomotor anchors. When interrogated in the multi-dimensional gradient space, we found that dopamine medication enhanced separation between functionally discrete sensory and higher-order networks. This increase in dispersion was dependent on an individual’s dopamine dose level, and increased dispersion was more apparent in regions enriched with dopamine receptor (DRD2) gene expression. Together, these findings substantiate a role for dopamine in modulating large-scale functional brain organisation. Our findings further confirm that medication targeting the dopamine system may achieve its benefit by restoring aspects of network topology, and suggest new hypotheses about how dopamine medication is influencing large-scale functional brain organisation in Parkinson’s disease.

## 1 Introduction

Neuromodulators shape and constrain neural activity, both at local circuits and at the level of large-scale networks. Our understanding of neuromodulation at the cellular level has outpaced our understanding of how neuromodulators regulate brain-wide activity (Roffman et al., 2016;Salvan et al., 2023), and, in turn, how they influence complex behaviours in health (O’Callaghan, Walpola, et al., 2021;Shine, Breakspear, et al., 2019) and disease (Orlando et al., 2023). Disease models offer unique insight into this problem. Here, we focus on Parkinson’s disease with its profound dopaminergic loss and pro-dopaminergic treatment strategies—offering a window into the effects of dopamine on large-scale brain organisation (Gratwicke et al., 2015;Meder et al., 2019).

Projecting from the substantia nigra and ventral tegmental area (VTA) in the midbrain, dopamine modulates firing patterns across the brain, particularly exerting influence upon the basal ganglia and frontal cortex (Camps et al., 1989;Hall et al., 1994). Dopamine acts on G-protein-coupled receptors, which can be segregated into the D1-type and D2-type receptor families that are broadly considered excitatory and inhibitory, respectively (Berke, 2018;Costa & Schoenbaum, 2022;Seamans & Yang, 2004). In Parkinson’s disease, the progressive death of dopamine-producing cells in the substantia nigra pars compacta disrupts ascending brainstem pathways, with significant consequences for whole-brain connectivity (Fiorenzato et al., 2019;Helmich et al., 2010;Kim et al., 2017;O’Callaghan et al., 2016). This dopaminergic degeneration predominately impacts the nigrostriatal tract (substantia nigra to striatum), affecting the mesocortical (VTA to cortex) and mesolimbic (VTA to limbic system) pathways to a lesser extent (Biondetti et al., 2021;Braak et al., 2004;Jellinger, 1991). And while dopamine is not the only ascending neuromodulator affected in Parkinson’s disease, functional MRI (fMRI) studies of dopamine withdrawal have helped to isolate potentially dopamine-specific mechanisms underlying network alterations in Parkinson’s disease (Aracil-Bolaños et al., 2021;Ballarini et al., 2018;Bell et al., 2015;Cai et al., 2022;Lewis et al., 2005;Ng et al., 2017;Shine, Bell, et al., 2019;Szewczyk-Krolikowski et al., 2014;Wang et al., 2022).

Dopamine replacement therapy in Parkinson’s disease is primarily targeted to address motor symptoms while its effects on cognition can be complex, with both deleterious and beneficial effects reported. Since the mesolimbic and mesocortical pathways are relatively spared in Parkinson’s disease, medication that works to boost global dopamine levels can ‘overdose’ this relatively intact ventral fronto-striatal circuitry (Aarts et al., 2014;Cools et al., 2001;Gotham et al., 1988;MacDonald et al., 2011;Swainson et al., 2000). Dopamine, like other monoaminergic and cholinergic neuromodulators, can be described by an inverted-U function, whereby too much or too little dopamine stimulation impairs behavioural performance (Arnsten, 1998;Cools, 2019;Goldman-Rakic, 1995;Robbins, 2000). This has implications for dopamine therapy, as a U-shaped dose-response curve means that systems where baseline dopamine is low will respond well to the drugs, and systems with intact/high dopamine levels will be impaired and pushed outside of their optimal range of function (Cools & D’Esposito, 2011;Rowe et al., 2008). Dopamine acts to modulate the signal-to-noise ratio of neural firing and consequently stabilises network dynamics (Guitart-Masip et al., 2016;Kroener et al., 2009;Seamans & Yang, 2004;Shafiei et al., 2019). Studies examining the interplay of functional connectivity and dopamine modulation (via pharmacological perturbation or receptor availability) highlight a role for dopamine in maintaining functional specialisation of networks (Korkki et al., 2025;Pedersen et al., 2023;Shafiei et al., 2019). Taken together, the differential effects of dopamine dysfunction and its treatment across the brain make Parkinson’s disease an important model to understand how dopamine modulates brain-wide organisation.

Dimensionality reduction techniques provide a means to characterise large-scale brain organisation using fMRI. For instance, using non-linear diffusion map embedding, we can model the brain as the superposition of eigenmodes describing axes of feature similarity between regions (Huntenburg et al., 2018;Margulies et al., 2016). Functional gradients that emerge from this approach reveal sensorimotor-to-associative and visual-to-somatomotor hierarchies of brain organisation, which are consistently observed across healthy people and patient cohorts (Bethlehem et al., 2020;Holmes et al., 2023;Hong et al., 2019;Huntenburg et al., 2017;Paquola et al., 2019;Tan et al., 2023). The so-called ‘principal’ sensorimotor-to-associative (or unimodal-to-transmodal) gradient—which emerges reliably from fMRI dimensionality map embedding—aligns closely with models of cortical hierarchy derived from cytoarchitecture and comparative anatomy (Burt et al., 2018;Fulcher et al., 2019;Mesulam, 1998;Shafiei et al., 2020;Vázquez-Rodríguez et al., 2019). Recent work has linked this gradient to the expression of dopamine D1 receptors in both humans and non-human primates (Froudist-Walsh et al., 2023;Pedersen et al., 2024), implicating dopamine in supporting this cortical architecture.

Here, we use gradient embedding approaches to characterise how cortical functional organisation is affected by dopamine in Parkinson’s disease, by examining resting-state scans acquired when individuals were both ‘on’ and ‘off’ their dopamine-replacement medication. Extending the typical gradients analyses, we explore the first three gradients in a multidimensional space using measures of Euclidean distance to quantify the dispersion*within*and*between*functional networks (Bethlehem et al., 2020). Given previous work showing enhanced network segregation in Parkinson’s disease on dopamine medication (Shine, Bell, et al., 2019), and the beneficial effects of increased network segregation seen in other neurodegenerative diseases of ageing (Ewers et al., 2021;He et al., 2023), we expected to find increased gradient dispersion in the on-dopamine state. We further determine whether changes in functional gradient organisation are related to individual medication dose and dopamine receptor gene expression. We hypothesised wide-spread changes in functional gradients in response to dopamine medication (compared to off medication), with more profound changes expected in regions that express a higher density of dopamine receptor and transporter genes.

## 2 Methods

### 2.1 Participants

Twenty-seven people with Parkinson’s disease were recruited from the Parkinson’s Disease Research Clinic at the Brain and Mind Centre, University of Sydney, Australia. All participants with Parkinson’s disease satisfied the United Kingdom Parkinson’s Disease Society Brain Bank criteria, did not meet Movement Disorders Society criteria for dementia (Martinez-Martin et al., 2011), and were aged between 50 and 80 years, with Hoehn and Yahr stages 1.5-3. All were taking anti-parkinsonian medications: six were on L-DOPA monotherapy; eight were on L-DOPA plus a dopaminergic agonist; four were on L-DOPA plus adjuvant therapy (rasagaline, entacapone or a monoamine oxidase inhibitor); a further four were on a combination of L-DOPA, dopaminergic agonist, and adjuvant therapy; one person was on dopaminergic agonist monotherapy; one was on an agonist plus adjuvant therapy; and three were on adjuvant therapy only. Dopaminergic dose equivalence (DDE) scores were calculated (Tomlinson et al., 2010) quantifying each person’s dopaminergic dose per day, irrespective of their particular medication regime. Nineteen age-, sex-, and education-matched controls were recruited via the clinic’s volunteer database. Control participants were screened for a history of neurological and psychiatric disorders, and none were using psychoactive medications. The study was approved by the local Ethics Committee, and all participants provided informed consent in accordance with the Declaration of Helsinki. SeeTable 1for demographic details and clinical characteristics.

**Table 1.** Demographics and clinical assessments of participants.

		PD	Controls	*p-* value
Age (years)		65.4 (8.63)	66.1 (11.70)	0.831
Education (years)		13.6 (2.89)	14.1 (2.76)	0.641
Male/female		17/9	8/11	0.221
MMSE		29.2 (1.29)	29.4 (0.92)	0.498
MoCA		27.1 (2.80)	27.6 (2.27)	0.427
Disease duration (years)		6.88 (5.48)		
DDE (mg/day)		680.28 (404.79)		
MDS-UPDRS III	PD-ON	29.67 (16.2)		**0.020**
	PD-OFF	36.19 (16.1)		
Hoehn and Yahr stage	PD-ON	2.19 (0.49)		0.107
	PD-OFF	2.48 (0.84)		

Data are presented as mean (SD). Comparisons of patient vs. control groups were performed with independent samples*t*-tests or contingency tables as appropriate; Parkinson’s disease ON vs. OFF comparisons were performed with paired samples*t*-tests.

MMSE = Mini-Mental State Examination; MoCA = Montreal Cognitive Assessment; DDE = Daily dopamine dose equivalent; MDS-UPDRS-III = Movement Disorders Society Unified Parkinson’s Disease Rating Scale part III.

Significant*p*-values (< 0.05) are bolded.

### 2.2 Study procedure

Participants with Parkinson’s disease were scanned on two occasions: i) ON their regular dopaminergic medications; and ii) OFF their regular dopaminergic medications following overnight withdrawal (i.e., 12–18 hours since last dose) with a mean of 5.02 (± 6.4 standard deviation) weeks between the sessions. At the ON visit, global cognition was assessed via the Mini-Mental State Examination (MMSE) (Folstein, 1983) and Montreal Cognitive Assessment (MoCA) (Nasreddine et al., 2005). At both ON and OFF visits, severity of motor symptoms was assessed using the Movement Disorder Society Revision of the Unified Parkinson’s Disease Rating Scale (MDS-UPDRS) Part III (Goetz et al., 2008). Control participants underwent MRI scans and cognitive testing at a single visit.

### 2.3 Image acquisition

Imaging was conducted on a 3-Tesla MRI scanner (GE medical systems). Whole brain T1-weighted structural images were acquired with sagittal orientation, 256 × 256 matrix, 200 slices, slice thickness of 1 mm, and echo time/repetition time = 2.7/7.2 ms. T2*-weighted echo-planar functional images were acquired with repetition time = 3 s, echo time = 36 ms, flip angle = 90º, 32 axial slices covering the whole brain, field of view = 220 mm, slice thickness of 3 mm, and raw voxel size = 3.9 mm × 3.9 mm × 4 mm. Each resting-state scan lasted 7 min (140 repetition times), and participants were instructed to lie awake with their eyes open.

### 2.4 Resting-state fMRI preprocessing and denoising

Preprocessing was completed using*fMRIPrep*20.2.3 (Esteban et al., 2019). Preprocessing involved segmentation and skullstripping, coregistration, normalisation, unwarping, noise component extraction, and finally subject-level removal of motion artefacts using automated ICA-AROMA (Pruim et al., 2015). SeeSupplementary Materialfor a full description of each step. The confounds timeseries data extracted from*fMRIprep*were passed through*fmridenoise*(Finc et al., 2019) specifying eight physiological signals to be regressed (mean physiological signals from white matter and cerebrospinal fluid, and their quadratic terms (Satterthwaite et al., 2013)). We applied a temporal bandpass filter (0.01 < f < 0.1 Hz).

### 2.5 Head motion

There were no significant differences in head movement, measured by mean framewise displacement, between healthy control subjects (mean = 0.176, SD = 0.117) and individuals with Parkinson’s disease (mean = 0.22, SD = 0.177;*p=*0.531). No difference was found in individuals with Parkinson’s disease across dopaminergic states (ON: mean = 0.229, SD = 0.196; OFF: mean = 0.201, SD = 0.158;*p*= 0.3693). We conducted post-hoc analysis to ensure our main findings were not confounded by head motion (seeSupplementary Material).

### 2.6 Generating functional gradients

Following preprocessing, the mean BOLD signal timeseries was extracted for 400 cortical regions using the Schaefer atlas (Schaefer et al., 2018) and z-scored using scripts adapted from the fieldtrip toolbox in MATLAB (Oostenveld et al., 2011). A 400 × 400 functional connectivity matrix was calculated for each individual using Pearson correlation. These 400 cortical regions were assigned to the seven Yeo functional networks (Yeo et al., 2011).

**Fig. 1.**
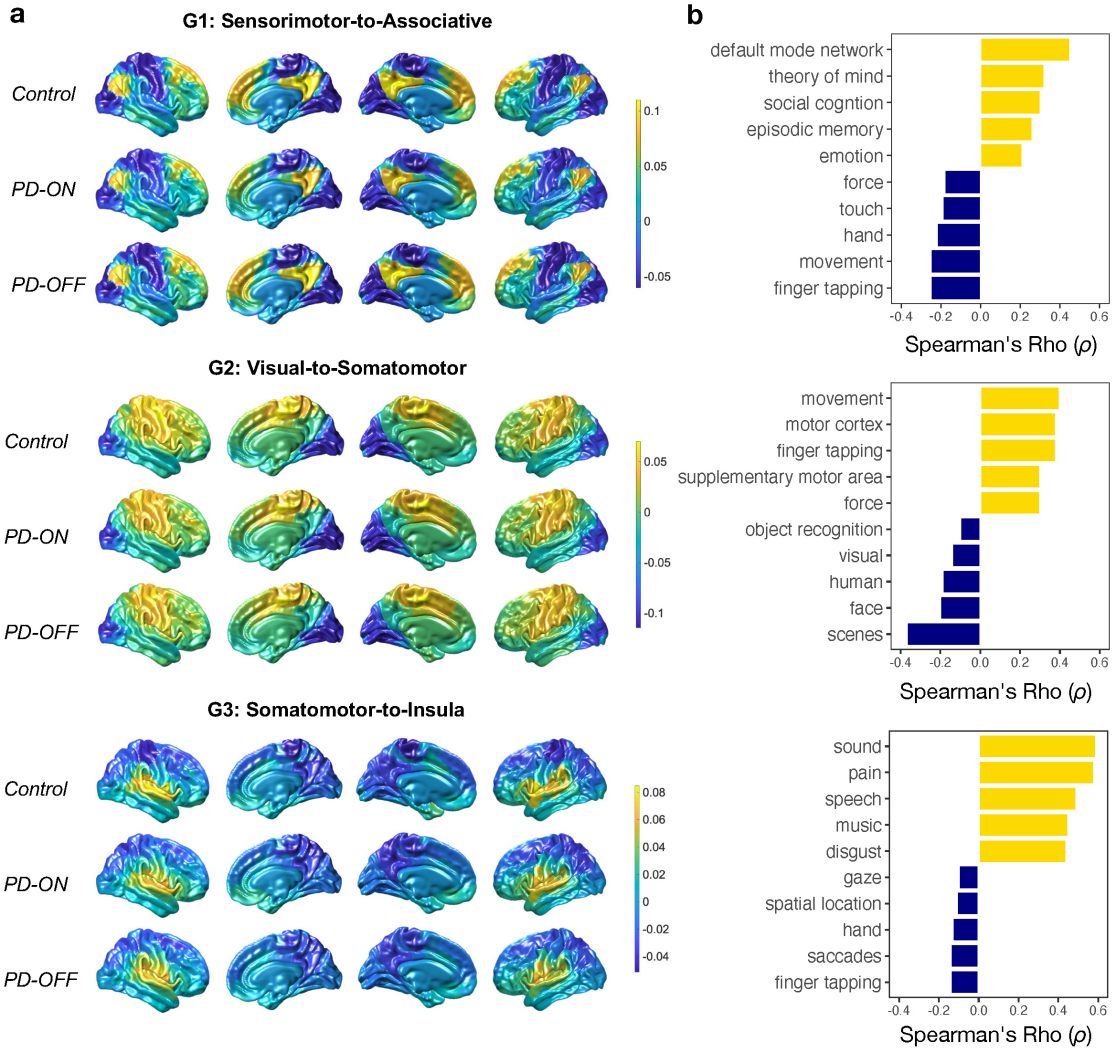
First three functional gradients and their associated functional correlates. (a) Group averages of the first three gradients. (b) Spearman correlation coefficients (ρ) between healthy control functional gradients and the five most positive and negative unique topics from 400 Neurosynth meta-analytic activation maps.

Functional gradients were generated using the*Brainspace*toolbox and custom MATLAB scripts (Vos de Wael et al., 2020). A population average functional connectivity matrix was calculated using the extracted timeseries data from all individuals (i.e., including all scans from the controls and Parkinson’s disease ON and OFF medication). This average matrix was thresholded, with the top 10% of measurements per row retained and all remaining measurements zeroed (Margulies et al., 2016;Vos de Wael et al., 2020). To express the similarity in connectivity profiles, the difference in angles between pairs of vectors from the functional connectivity matrix was calculated using a cosine similarity function. This resulting cosine similarity matrix denotes the similarity between each possible pair of regions. We then applied diffusion map embedding, that is, a nonlinear manifold learning approach to dimensionality reduction, to the affinity matrix to identify components explaining variance in descending order. The algorithm is governed by a single parameter α, which controls the influence of density of sampling points on the manifold (α = 0, maximal influence; α = 1, no influence). In line with previous studies and to preserve the global relationships between data points in the embedded space, we set α = 0.5 (Hong et al., 2019;Langs et al., 2015;Vos de Wael et al., 2020). We generated group-level gradient components from the average functional connectivity matrices of all participants. Individual gradient components were then aligned to the group-level templates using Procrustes alignment (Langs et al., 2015). Average gradient components for each group, that is, PD-ON, PD-OFF, and controls, were then mapped back onto the cortical surface (Fig. 1a). For each gradient, we calculated the variance explained by dividing the gradient’s eigenvalue with the sum of the eigenvalues for all gradients.

FollowingBethlehem et al. (2020), we conducted analyses on the first three gradients, which in our cohort accounted for >35% of variance and individually each explained >10% variance. The three gradients were labelled according to established conventions (Bethlehem et al., 2020;Margulies et al., 2016;Vos de Wael et al., 2020): Gradient 1 (G1), anchored by a sensorimotor-to-association axis (the so-called ‘principal gradient’); Gradient 2 (G2) visual-to-somatomotor; and Gradient 3 (G3) somatomotor-to-insula. While G1 did not explain the most variance (i.e., variance explained by G1 = 12.53%; G2 = 14.23%; G3 = 10.17%), we confirmed that it correlated significantly with the principal gradient (Margulies et al., 2016) and with presumed network hierarchies (Tan et al., 2023;Zarkali et al., 2021), establishing it as the commonly observed sensorimotor-to-associative principal gradient (SeeSupplementary Material). We also confirmed that the order of variance explained by G1, G2, and G3 did not change when gradients were generated without the controls (SeeSupplementary Material).

To cross-validate the axes of the three gradients maps, we compared them against cognitive terms defined by probabilistic functional association maps from the Neurosynth database. We used the gradients from the healthy controls for this validation step. Using the Neuroimaging Meta-Analysis Research Environment (NiMARE) (Salo et al., 2023), a set of 400 topics containing terms clustered by a latent Dirichlet allocation (LDA) topic model were extracted from the abstracts of all articles in the Neurosynth database (Version 7) (Yarkoni et al., 2011). The coordinates were parcellated into 400 cortical regions (Schaefer et al., 2018), and probabilistic time series maps for each term were extracted. Topics were matched with abstracts that contained relevant terms. Then, coordinate-based meta-analyses were performed using multiple kernel density analysis. Statistical significance was calculated, and corrections for multiple comparisons were conducted using Monte Carlo simulations. Together, this outputs a probabilistic measure of how regional activation patterns are related to cognitive processes (seeSupplementary Materialfor a full list of topic terms). We then used Spearman correlation to identify associations between the 400 meta-analytic activation maps and the first three gradients of the control group, in order to establish probabilistic term associations. We focus on the correlation values as a ranking measure, in order to identity the top associated terms, and do not assess their statistical significance. We present the five strongest positive and negative terms correlated with each gradient (Fig. 1b).

### 2.7 Functional gradients analyses

While the 19 healthy controls were included to support the group-level gradient template construction, and to confirm that a similar overall gradient macrostructure was present across both the groups, the following analyses were restricted to our comparison of interest: Parkinson’s disease patients ON and OFF their dopamine medication.

#### 2.7.1 Regional and network differences

We compared differences in gradient score at both the regional and network level using non-parametric, paired permutation between Parkinson’s disease patients ON (PD-ON) and OFF (PD-OFF) dopamine medication.

#### 2.7.2 Dispersion of functional gradients in multidimensional space

FollowingBethlehem et al. (2020), we constructed a manifold space comprising the first three cortical gradients. This captures the hierarchical organisation and differentiation of functional networks within three-dimensional space. To quantify the three-dimensional manifold, we calculated dispersion measures within and between the seven networks (Bethlehem et al., 2020). For each individual, we calculated*between network dispersion*, that is, the Euclidean distance between the centres (median value) of each network in manifold space, and*within network dispersion*, that is, the sum of squared Euclidean distances of nodes in an individual’s network to their network centre. To compare dispersion measures between the groups (i.e., PD-ON vs. PD-OFF), we conducted non-parametric permutation testing (1000 permutations), which provides a control for family-wise error rate (FWE), offering a means of error control and improved power for small sample neuroimaging studies by directly accounting for multiple comparisons through the generation of a maximum statistic under the null distribution (Camargo et al., 2008;Lindquist & Mejia, 2015;Nichols & Holmes, 2001).

We then determined whether significant changes in dispersion metrics that were identified ON vs. OFF dopamine would depend on an individual’s dopamine dose. To test this, dopamine dose equivalence (DDE) scores were entered as an interaction term in linear mixed models, that is, dispersion metric ~ dopamine state (ON vs OFF) × DDE + (1 | subject), with participant entered as a random effect to account for repeated measures. The models were implemented in R (version 4.2.1,R Core Team, 2022) using the*afex*(Singmann et al., 2020) and*lmerTest*packages (Kuznetsova et al., 2017).

### 2.8 Dopamine receptor and transporter gene expression

To test our hypothesis that dispersion changes ON vs. OFF medication would relate to neuromodulatory receptor distribution, we used gene expression data obtained from the Allen Human Brain Atlas (AHBA) (Hawrylycz et al., 2012,^2015^). Expression profiles for genes of dopaminergic receptors (DRD1, DRD2, DRD3, DRD4, DRD5) and monoamine transporters (DAT, NET, SERT) were preselected. We conducted preprocessing following recommended methodology (Arnatkevic̆iūtė et al., 2019,^2023^;Fornito et al., 2019) using the Python toolbox*abagen*(Markello et al., 2021). Steps involved reannotation of microarray probes and filtering for probes where expression measures were above a background threshold in more than 50% of samples across donors (n = 6). A representative probe for a gene was selected based on highest differential stability among donors. Probes were then aggregated across donors and gene expression data were normalised across the cortex using scaled, outlier-robust sigmoid normalisation. Data were then registered to standard MNI space, allowing for the mapping of the tissue samples from AHBA to the 400 cortical regions used in the gradient analysis conducted in the present study. A subset of 15,631 genes (of 20,737 initially included in the Allen atlas gene expression data) survived these preprocessing and quality assurance steps, including four of our preselected dopaminergic receptor and transporter genes (i.e., DRD1, DRD2, DRD4, and DAT). Expression profiles for those four genes were extracted per region and z-score normalised. Spin permutation tests (1000 iterations) of Spearman correlations were performed between regional gene expression and the average change in gradient score in patients ON vs. OFF dopamine medication (denoted as Δ).

## 3 Results

### 3.1 Demographic and clinical characteristics

Parkinson’s disease and control groups were well matched on demographics and cognitive screens (Table 1). As expected, Parkinson’s disease OFF their dopamine medication, compared to ON, showed a significant increase in their MDS-UPDRS-III scores (F(1, 21.28) = 6.34, p = 0.020).

### 3.2 Characterising functional gradient organisation

The first three gradients showed functional differentiation running from sensorimotor-to-association (G1), visual-to-somatomotor (G2), and somatomotor-to-insula (G3) (Fig. 1a). G1 was anchored by primary sensory cortices at the lower end and associative, default mode cortical areas at the upper end—representing a sensorimotor-associative, or unimodal-transmodal axis. G2 was anchored by the visual cortex at the lower end and the primary motor cortex at the upper end. G3 was anchored by insula at the lower end and the primary motor area at the higher end. These gradients were associated with*Neurosynth*cognitive topics (Fig. 1b) that were consistent with functions related to the ends of the three axes described above. The underlying shape of the first three gradients was relatively stable across groups. Post-hoc analyses compared summary measures of the gradient distribution (i.e., the range of the gradient distribution and its bimodality) across groups, and no significant differences emerged (seeSupplementary Material).

### 3.3 Regional gradient differences across dopamine medication states

Differences in regional gradient score were distributed across the cortex for PD-ON vs. PD-OFF, as shown inFigure 2lower panel. We found 19 significantly different regions in G1 (*p*< 0.05), 36 significantly different regions in the G2 (*p*< 0.05), and 28 different regions in G3 (*p*< 0.05). SeeSupplementary Materialfor a full description of the regions.

**Fig. 2.**
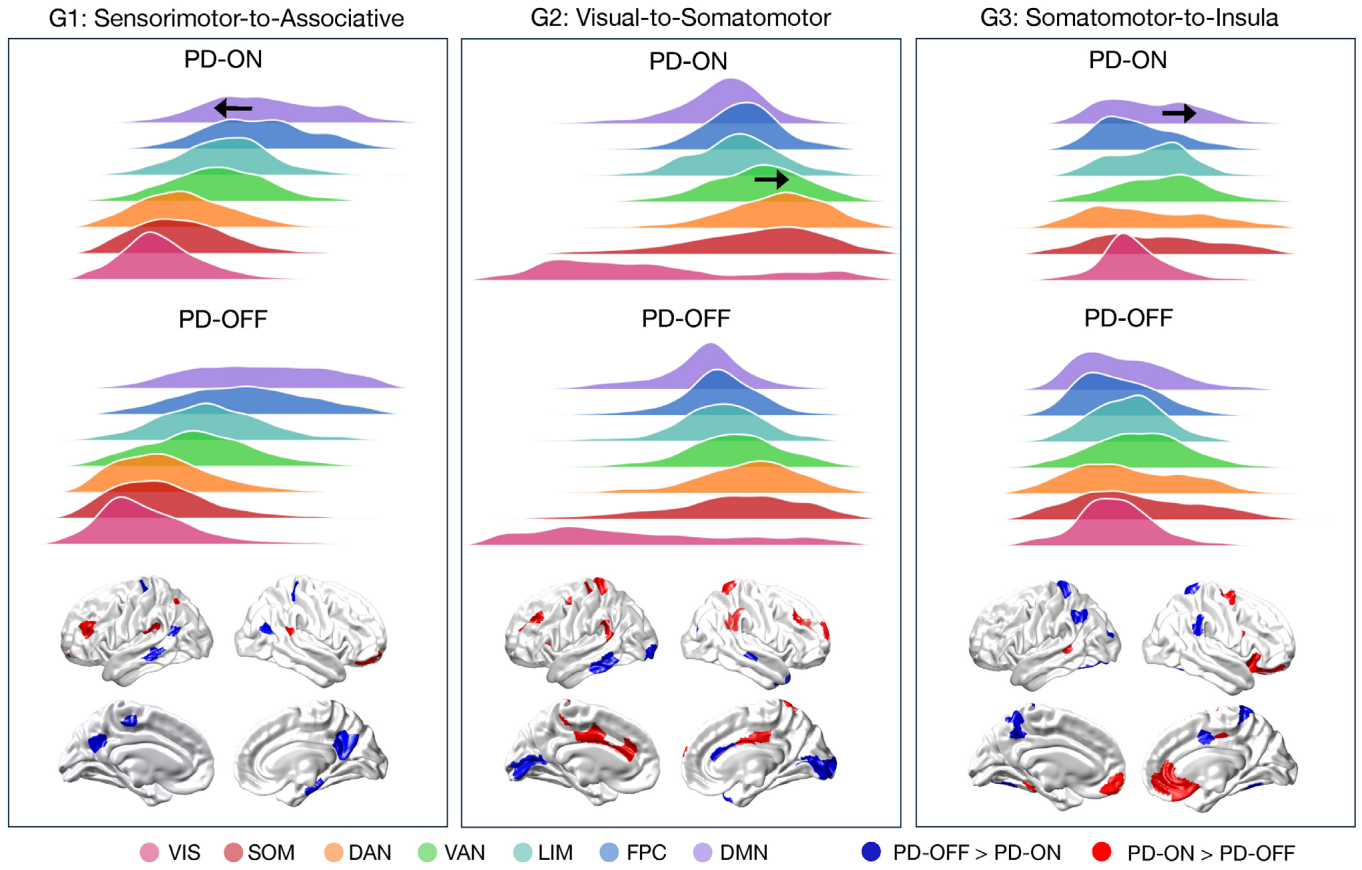
Regional and network-level changes in gradients for Parkinson’s disease ON vs. OFF dopamine medication. The upper panel shows average gradient score distributions across the functional networks for the first three gradients between PD-ON and PD-OFF groups. Bolded arrows show the direction of significant network shifts in the ON dopamine state, relative to OFF dopamine. The lower panel shows significant regional differences in gradient scores between PD-ON and PD-OFF, tested using permutation paired t-tests with Blue: PD-OFF > PD-ON = t statistic was higher for PD-OFF; Red: PD-ON > PD-OFF = t statistic was higher for PD-ON. Functional networks: DAN = dorsal attention; DMN = default mode; FPC = frontoparietal control; LIM = limbic; SOM = somatomotor; VAN = ventral attention; VIS = visual.

### 3.4 Network gradient differences across dopamine medication states

We compared the average gradient score at the network level between PD-ON vs. PD-OFF, as shown in the upper panel ofFigure 2. For G1, the default mode network was significantly shifted in patients ON dopamine medication, such that the mean was closer to the sensorimotor anchor (t = -2.28,*p*= 0.038). The ventral attention network of G2 shifted significantly towards the somatomotor anchor in patients ON dopamine compared to OFF (t = 2.26,*p*= 0.034). In G3, the default mode network was also shifted towards the somatomotor anchor in the ON state (t = 2.17,*p*= 0.040). We found no evidence for a correlation between the magnitude of network shifts and changes in head motion over the scans (seeSupplementary Material). Consistent with these network differences, when comparing the overall shape of the gradient distributions via non-parametric Kolmogorov-Smirnov tests, we found significant differences for each gradient ON vs. OFF medication (G1:*D*= 0.032,*p*_FDR_< 0.001; G2:*D*= 0.026,*p*_FDR_= 0.002; G3:*D*= 0.029,*p*_FDR_< 0.001).

### 3.5 Differences in multidimensional gradient dispersion across dopamine medication states

**Fig. 3.**
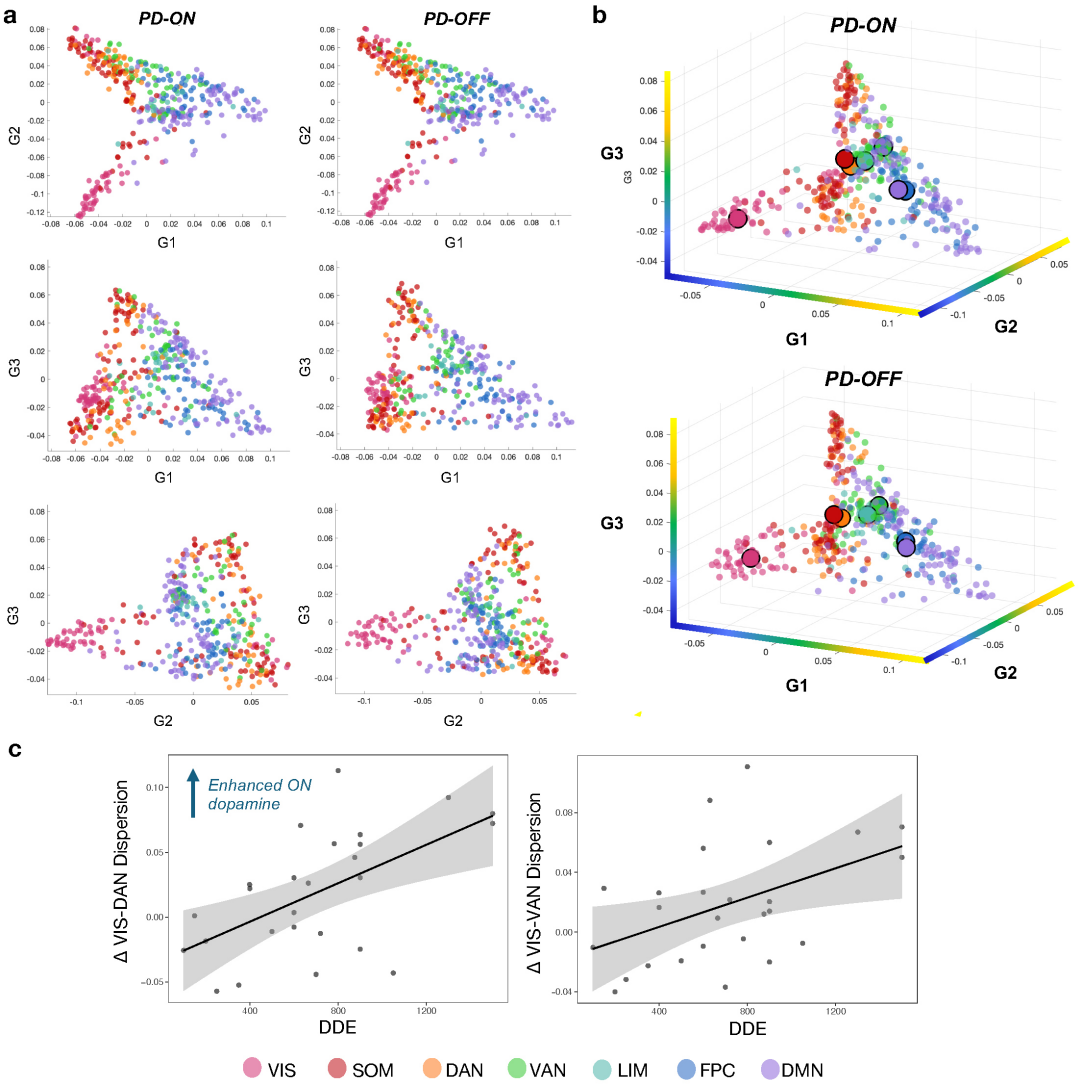
Functional gradients on and off dopamine medication in two- and three-dimensional space. (a) Average gradient scores for PD-ON and PD-OFF for the first three gradients in two-dimensional space, coloured by functional network assignment. (b) First three gradients in three-dimensional space for PD-ON and PD-OFF, coloured by functional network assignment; here the larger, outlined points indicate the group-level network centroids. The colour bars along the axes represent the cortical gradient surface (yellow = positive, blue = negative value along individual gradients). (c) Relationship between dopamine dose equivalent scores (DDE mg/day) with the drug-induced change in significant network dispersion. The change (Δ) calculated as dispersion ON*minus*dispersion OFF. Functional networks: DAN = dorsal attention; DMN = default mode; FPC = frontoparietal control; LIM = limbic; SOM = somatomotor; VAN = ventral attention; VIS = visual.

We characterised differences in functional gradient organisation across dopamine states by projecting brain regions into a space made of the first three gradient axes. For simplicity, we present the relationships between each pair of gradients in a two-dimensional gradient space (Fig. 3a), followed by the gradients projected into the three-dimensional space (Fig. 3b). To quantify changes in this three-dimensional space, we used previously defined dispersion metrics (Bethlehem et al., 2020), whereby a higher dispersion value signifies greater functional differentiation across regions within a network, or between two networks. Within-network dispersion did not change ON vs. OFF dopamine medication (*p*> 0.05). In contrast, we found evidence for between-network dispersion differences in the ON state compared to OFF, with distance between the visual–dorsal attention (t = 2.224,*p*= 0.033) and visual–ventral attention (t = 2.205,*p*= 0.038) networks showing significantly higher dispersion in the Parkinson’s disease ON medication state.

### 3.6 Dopamine dose interactions with measures of dispersion

For the dispersion metrics that emerged as significantly different between the groups (i.e., visual–dorsal attention and visual–ventral attention), we tested whether these changes related to individual levels of dopamine dose. Significant interactions were observed between DDE and dispersion between the visual–dorsal attention networks (F(1, 24) = 12.23,*p*_FDR_= 0.004) and visual–ventral attention networks (F(1, 24) = 6.47,*p*_FDR_= 0.018). Together, suggesting that the magnitude of dispersion changes ON medication differed based on an individual’s daily dopamine dose. To visualise the directionality of this relationship,Figure 3cplots each individual’s change in network dispersion metrics (i.e., ON*minus*OFF) against their daily dopamine dose level. The results confirm that higher dopamine dose levels were associated with greater increases in dispersion in the ON medication state. The interaction did not qualitatively change when age and disease severity (measured by off-state UPDRS-III) were included as covariates, as model selection procedures indicated that these covariates did not significantly improve the model fit (seeSupplementary Material). When controlling for head motion in our model, the interaction effect did not qualitatively change, nor did it contribute as a main effect (seeSupplementary Material).

### 3.7 Gene expression links with changes in functional gradients

**Fig. 4.**
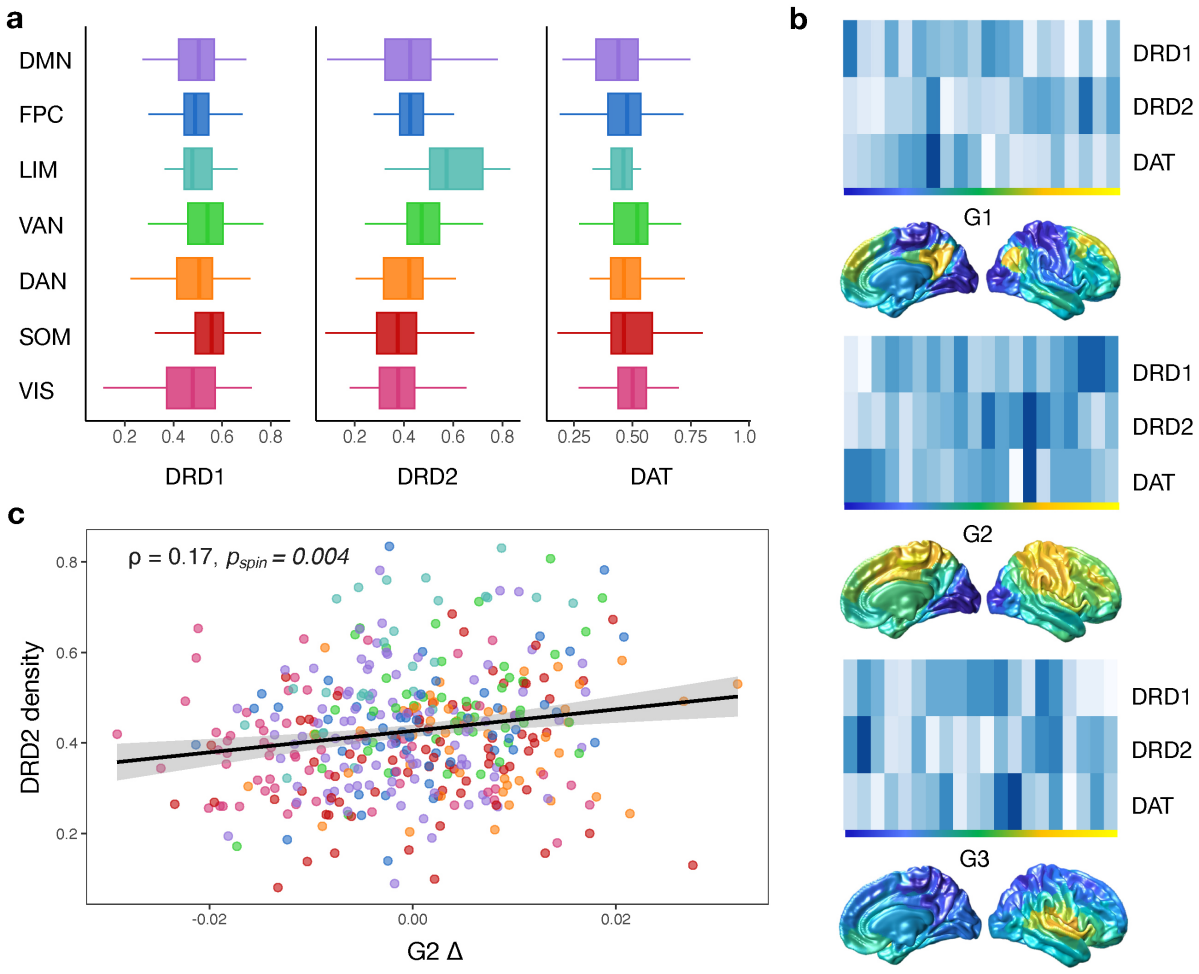
Dopamine receptor gradients. (a) Receptor densities, as extracted from the Allen Brain, plotted across each functional network. (b) Distribution of DRD1, DRD2 and DAT averaged across percentile bins for the first three gradients of the PD-ON group (Navy blue = high density, white = low density), with each functional gradient projected onto the cortical surface next to the corresponding axis. (c) Correlating G2 changes (PD-ON*minus*PD-OFF; Δ) with the density of DRD2 receptor expression across the cortex (Spearman*ρ*= 0.17,*p*_FDR_= 0.003,*p*_spin_= 0.004). Functional networks: DAN = dorsal attention; DMN = default mode; FPC = frontoparietal control; LIM = limbic; SOM = somatomotor; VAN = ventral attention; VIS = visual.

We examined whether the changes observed in gradient organisation were related to the gene-expression distribution of dopaminergic receptors using the AHBA receptor expression maps for genes encoding DRD1, DRD2, and the dopamine transporter (DAT). For visualisation purposes, we show the distribution of the extracted gene expression maps across the functional network hierarchy (Fig. 4a) and organised with respect to the first three gradients in the PD-ON group (Fig. 4b). We found a significant correlation between the change in G2 score (i.e., PD-ON*minus*PD-OFF) and the density of DRD2 receptors (Spearman*ρ*= 0.17,*p*_FDR_= 0.003,*p*_spin_= 0.004;Fig. 4c). This relationship was replicated with the D2 receptor expression density obtained from*neuromaps*PET data (seeSupplementary Material) (Markello et al., 2022).

## 4 Discussion

Here, we demonstrate evidence for dopamine-related changes in the organisation of functional gradients in Parkinson’s disease. In individuals ON their dopamine medication, relative to OFF, we found shifts in higher-order networks towards somatomotor anchors along single gradients. In a multidimensional gradient space, our findings were consistent with dopamine medication increasing dispersion between functionally discrete networks. This finding was substantiated by a dose-dependent relationship, whereby higher dopamine medication doses were associated with greater increases in the network distance between sensory and higher order networks. A dopaminergic link was further evidenced as the extent of changes in the visual-to-somatomotor functional gradient across medication states was related to D2 receptor expression patterns. Together, our results reveal new insights into how dopamine medication in Parkinson’s disease reconfigures network organisation, emphasising a key role for dopamine modulation of network-level brain organisation.

Our findings confirm that the overall macroscale ordering of functional gradients was preserved ON vs. OFF dopamine medication (Fig. 1a); however, differences emerged at the uni- and multi-dimensional levels to reveal the effects of dopamine on gradient organisation. Importantly, on-medication changes were apparent across the first three gradients examined, supporting the utility of viewing these in a multidimensional space that simultaneously describes how gradients may change in relation to each other (Bethlehem et al., 2020). A broadly consistent pattern of changes at the unidimensional level was a shift in higher-order networks (i.e., default mode and ventral attention) towards somatomotor anchors in the ON dopamine state. As such, at the single-gradient level, those higher-order networks became more functionally similar to somatomotor networks. One interpretation of this is the possibility that the substantial improvements in motor symptoms typically seen with dopamine-replacement therapy may rely on a re-deployment, as it were, of more brain networks in the service of sensorimotor function. This idea harks back to the concept of redundancy in biological networks (Glassman, 1987;Tononi et al., 1999), where alternate routes to a given brain function might be employed in the face of insult or pathology, providing a means of*resilience*or*reserve*(Stanford et al., 2024;Stern, 2002).

Yet, results from the unidimensional, single-gradient level mapping techniques do not disambiguate within- and between-changes in functional communities (Bethlehem et al., 2020). When we examined within- and between-network dispersion changes, we found that between-network dispersion was increased on dopamine medication between the visual network to both the ventral and dorsal attention networks, and no within-network differences were observed. Therefore, in the multidimensional space, dopamine medication reinstated a more segregated organisation between primary sensory visual networks and higher-order attentional networks. Increasing functional differentiation between select networks can be advantageous in the context of neurodegenerative diseases of ageing, with a more modular organisation linked to better preserved cognitive abilities (Ewers et al., 2021). Indeed, gradients-based measures have shown that reduced dispersion in Alzheimer’s disease is associated with worse cognitive performance (He et al., 2023), and reduced separation between primary visual regions and higher-order attentional networks has been implicated in Parkinson’s disease visual hallucinations (Tan et al., 2023) and in schizophrenia (Holmes et al., 2023). Taken together, our results raise the possibility that pro-cognitive effects of dopamine replacement in Parkinson’s disease may stem, at least in part, from an improved differentiation between primary sensory and attentional networks.

Our results highlight a difference in the effect of dopamine medication at the single-gradient level versus the multidimensional level. With individual gradients showing higher order networks shifting towards the sensorimotor anchors on dopamine (i.e., integrating); while in the shared gradient space we observed greater dispersion between higher-order and sensorimotor networks on dopamine (i.e., segregating). Literature examining network-level and whole-brain topology in Parkinson’s disease is varied—that is, there is a mix of findings across increased versus decreased connectivity, and increased versus decreased integration/segregation. These heterogenous results are influenced by patients’ disease stage, their clinical phenotype, and their medication state. For instance, both increased and decreased connectivity between individual networks—measured during resting-state fMRI—have been associated with non-motor symptoms in Parkinson’s disease (Boon et al., 2020;Shine et al., 2015;Walpola et al., 2020). Meanwhile, whole-brain measures of brain topology, including changes in the balance between integration and segregation, have also been shown in Parkinson’s disease (Kim et al., 2017;Shine, Bell, et al., 2019), with different patterns of integration-segregation changes related to motor and non-motor symptoms (Dirkx et al., 2023;Ehgoetz Martens et al., 2018;Zarkali et al., 2022). Our findings emphasise that such varied effects of integration versus segregation may be observed within a single cohort, by looking across single- versus multi-dimensional gradient levels. A compression of the ‘principal’ sensorimotor-to-associative gradient has previously been reported in Parkinson’s disease (Tan et al., 2023), and our results show that changes in single gradients are sensitive to medication effects. While ours is the first to compare gradients in a multidimensional approach ON vs. OFF dopamine medication, previous work shows greater network-level segregation in the on-dopamine state compared to the off-dopamine state (Shine, Bell, et al., 2019). Given the conceptual overlap between multidimensional gradient dispersion and graph theoretic measures of network segregation and modularity (Bethlehem et al., 2020;He et al., 2023), these previous findings are consistent with the increased network dispersion we show here in the on-dopamine state.

The change in dispersion on dopamine medication showed a dose-dependent relationship, with individuals on higher daily doses of dopamine showing a greater increase in dispersion. To further complement a dopaminergic interpretation of our results, we found that the extent of change in the visual-to-somatomotor gradient (G2) between medication relates to densities of D2 receptor gene expression. We replicated this relationship using D2 receptor densities obtained from normative PET maps (Markello et al., 2022), however future work would ideally extend these findings by using PET data obtained from the subjects themselves. Although indirect, this evidence further supports the idea that variations in receptor density are likely to be an important determinant of how large-scale brain organisation reconfigures in response to psychoactive drugs (Luppi et al., 2023). Previous work established an association between D1 receptor distribution and the sensorimotor-to-associative gradient of cortical organisation (Froudist-Walsh et al., 2023;Pedersen et al., 2024). In contrast, our findings did not show an association between D1 receptor expression and the principal gradient, noting that previous work was in normative cohorts raising the possibility that different receptor-gradients associations could reflect disease effects. We instead identify a novel association: that changes in visual-to-somatomotor gradient organisation, in response to elevating dopaminergic levels with medication, may be constrained by D2 expression. Our select finding of D2 involvement here may be driven by D2 showing a much lower concentration across the cortex (compared to D1), but relatively higher concentrations in the temporal and visual cortex (Camps et al., 1989;Hall et al., 1994;Hansen et al., 2022;Palomero-Gallagher & Zilles, 2018). Furthermore, receptor activation is not solely dependent on ligand availability, but is determined by the dynamics and binding properties of those receptors. D1 and D2 receptor types show different temporal and affinity profiles (Dreyer et al., 2010;Liu et al., 2021). Dopaminergic neurons exhibit dominant low-frequency tonic firing patterns along with intermittent phasic bursts at basal levels (Venton et al., 2003). Under phasic firing patterns, D1 receptors, which are low affinity, are preferentially occupied while D2 receptors, which are of high affinity, are thus more sensitive to changes in tonic dopamine levels (Dreyer et al., 2010). This may help explain an outsized role for D2 receptors, if they remain preferentially engaged under conditions of sustained, elevated dopamine levels that have a reduced dynamic, phasic range—conditions that may occur under antiparkinsonian drugs, consistent with models of what occurs under cholinesterase inhibitors (Dumas & Newhouse, 2011).

While extensive literature has established the role of dopamine in the basal ganglia (Keeler et al., 2014), the effect of dopamine upon large-scale cortical networks, and its relationship to different dopamine receptor families, has received limited attention (Roffman et al., 2016;Shafiei et al., 2019). A dual-state theory of prefrontal dopamine function describes the differential activation of dopamine receptors to two discrete dynamical regimes (Durstewitz & Seamans, 2008;Seamans & Robbins, 2010;Seamans & Yang, 2004). A state in which D1 receptor activation dominates arises at moderate concentrations and facilitates a high energy barrier, or network stability, favouring maintenance of cognitive operations. Meanwhile, a D2-dominated state occurs at either very high or low dopamine concentrations and facilitates a low energy barrier, thus engaging cognitive flexibility and the shifting between network states. These dopamine-mediated brain states help determine reconfigurations in network topology that are critical for adaptive cognition (Roffman et al., 2016) and are altered in neuropsychiatric conditions where the dopamine system is affected (Braun et al., 2021;Durstewitz et al., 2021). Our results provide further evidence, from a Parkinson’s disease model, that dopamine may alter network topology in a number of ways, which relate, in part, to the expression of dopamine receptors. Here, we focus on cortical network topography. However, given the striatal changes that occur in Parkinson’s disease, replicating this work to characterise striatal gradients would be of interest—particularly given evidence of a link between striatal gradients and dopamine transporter availability in Parkinson’s disease (Oldehinkel et al., 2022).

Aside from a fundamental need to progress understanding of dopamine—and other neuromodulators—effects upon large-scale brain organisation, these questions have clinical relevance. Preservation and adaptation of brain network topology has emerged as a defining trait of cognitive resilience in the face of pathology (Ewers et al., 2021;Rittman et al., 2019). The drugs we give people across neuropsychiatric illnesses have the potential for wide-spread, yet nuanced, effects upon these topologies, mimicking their endogenous ligands (Shine, 2023). In neurodegenerative diseases of ageing, as efforts continue towards a precision medicine that is informed by neuroimaging (Cope et al., 2021;Hezemans et al., 2022;O’Callaghan, Hezemans, et al., 2021;Zarkali et al., 2024), there is an urgent need to reconcile the effects of psychoactive drugs on large-scale brain organisation, while charting their beneficial (and possibly deleterious) effects on behaviour. In the current study, we have not addressed disease-state effects (i.e., comparisons between controls and people with Parkinson’s disease), nor have we addressed whether medication effects on brain topology resemble more (or less) the patterns seen in controls. Noting that in Parkinson’s disease, previous work indicates that dopaminergic medication effects do not simply ‘normalise’ resting-state connectivity patterns with respect to controls (Ng et al., 2017). Future studies are needed to more fully characterise the clinical implications of medication-related effects on low-dimensional, large-scale brain organisation. Our work and others’ confirms that gradients-based analyses may be a useful lens to approach this problem in Parkinson’s disease and related disorders (He et al., 2023;Tan et al., 2023;Zarkali et al., 2021).

In summary, we used dimensionality reduction techniques to reveal dopaminergic involvement underlying the organisation of functional gradients in Parkinson’s disease. Using resting state fMRI under a pharmacological manipulation, we show that macroscopic reorganisation of networks in a three-dimensional gradient space is dose dependent and related to the expression of dopamine receptors. Together, these findings further substantiate a role for dopamine in modulating large-scale functional brain organisation. Medication that targets these systems may achieve their benefit by restoring aspects of topological network organisation.

## Supplementary Material

Supplementary Material

## Data Availability

Data and code to reproduce manuscript figures and statistical analyses are available through the Open Science Framework (https://osf.io/cf4m2/). The*AHBA*is available athttps://human.brain-map.org/static/download. The*Neurosynth*database is available athttps://neurosynth.org/.
