# Supplementary material for: Dopamine alters functional gradients in Parkinson’s disease
Source: Imaging Neurosci (Camb). 2025 May 6;3:imag_a_00564. doi: 10.1162/imag_a_00564 (PMC12319910; doi:10.1162/imag_a_00564)
Supplement: Supplementary Material [file imag_a_00564-supp.pdf]

<u>Supplementary Material</u>

Dopamine alters functional gradients in Parkinson's disease

1. fMRI preprocessing	Page 2
2. Gradient 1 (principal gradient) validation	Page 5
3. Alternative group-level gradient generation	Page 6
4. Gradient kurtosis, skew, range and bimodality	Page 8
5. List of regions with significantly different gradient scores	Page 11
6. Subject level gradients in three-dimensional space	Page 14
7. Selection of explanatory variables for dispersion models	Page 15
8. Testing motion as a confounding variable in our models	Page 17
9. Validating relationship between D2 receptor density and change in Gradient 2	Page 18
10. Top 3 terms of each LDA-400 topic from the Neurosynth metanalysis	Page 19
11. References	Page 31

1. fMRI preprocessing

Anatomical data preprocessing

The T1-weighted (T1w) image was corrected for intensity non-uniformity (INU) with N4BiasFieldCorrection (Tustison et al., 2010), distributed with ANTs 2.3.3 ((Avants et al., 2008), RRID:SCR_004757)), and used as T1w-reference throughout the workflow. The T1w reference was then skull-stripped with a Nipype implementation of the antsBrainExtraction.sh workflow (from ANTs), using OASIS30ANTs as target template. Brain tissue segmentation of cerebrospinal fluid (CSF), white-matter (WM) and gray-matter (GM) was performed on the brain-extracted T1w using fast (FSL 5.0.9, RRID:SCR_002823, (Zhang et al., 2001)). Volume-based spatial normalisation to two standard spaces (MNI152NLin2009cAsym, MNI152NLin6Asym) was performed through nonlinear registration with antsRegistration (ANTs 2.3.3), using brain-extracted versions of both T1w reference and the T1w template. The following templates were selected for spatial normalisation: ICBM 152 Nonlinear Asymmetrical template version 2009c [(Fonov et al., 2009), RRID:SCR_008796; TemplateFlow ID: MNI152NLin2009cAsym], FSL's MNI ICBM 152 non-linear 6th Generation Asymmetric Average Brain Stereotaxic Registration Model [(Evans et al., 2012)RRID:SCR_002823; TemplateFlow ID: MNI152NLin6Asym].

Functional data preprocessing

First, a reference volume and its skull-stripped version were generated using a custom methodology of fMRIPrep (Esteban et al., 2019). Susceptibility distortion correction (SDC) was omitted. The BOLD reference was then co-registered to the T1w reference using flirt (FSL 5.0.9, (Jenkinson & Smith, 2001)) with the boundary-based registration (Greve & Fischl, 2009) cost-function. Coregistration was configured with nine degrees of freedom to account for distortions remaining in the BOLD reference. Head-motion parameters with respect to the BOLD reference (transformation matrices, and six corresponding rotation and translation parameters) are estimated before any spatiotemporal filtering using mcflirt (FSL 5.0.9, (Jenkinson et al., 2002)). The BOLD time-series (including slice-timing correction when applied) were resampled onto their original, native space by applying the transforms to correct for head-motion. These resampled BOLD time-series will be referred to as preprocessed BOLD in original space, or just preprocessed BOLD. The BOLD time-series were resampled into standard space, generating a preprocessed BOLD run in MNI152NLin2009cAsym space. Automatic removal of motion artifacts using independent component analysis (ICA-AROMA,

(Pruim et al., 2015)) was performed on the preprocessed BOLD on MNI space time-series after removal of non-steady state volumes and spatial smoothing with an isotropic, Gaussian kernel of 6mm FWHM (fullwidth half-maximum). Corresponding "non-aggresively" denoised runs were produced after such smoothing. Additionally, the "aggressive" noise-regressors were collected and placed in the corresponding confounds file.

Several confounding time-series were calculated based on the preprocessed BOLD: framewise displacement (FD), DVARS and three region-wise global signals. FD was computed using two formulations following Power (absolute sum of relative motions, (Power et al., 2014)) and Jenkinson (relative root mean square displacement between affines, (Jenkinson et al., 2002)). FD and DVARS are calculated for each functional run, both using their implementations in Nipype (following the definitions by Power et al. 2014). The three global signals are extracted within the CSF, the WM, and the whole-brain masks. Additionally, a set of physiological regressors were extracted to allow for component-based noise correction (CompCor, (Behzadi et al., 2007)). Principal components are estimated after high-pass filtering the preprocessed BOLD time-series (using a discrete cosine filter with 128s cut-off) for the two CompCor variants: temporal (tCompCor) and anatomical (aCompCor). tCompCor components are then calculated from the top 2% variable voxels within the brain mask. For aCompCor, three probabilistic masks (CSF, WM and combined CSF+WM) are generated in anatomical space. The implementation differs from that of Behzadi et al. in that instead of eroding the masks by 2 pixels on BOLD space, the aCompCor masks are subtracted a mask of pixels that likely contain a volume fraction of GM. This mask is obtained by thresholding the corresponding partial volume map at 0.05, and it ensures components are not extracted from voxels containing a minimal fraction of GM.

Finally, these masks were resampled into BOLD space and binarised by thresholding at 0.99 (as in the original implementation). Components were also calculated separately within the WM and CSF masks. For each CompCor decomposition, the k components with the largest singular values are retained, such that the retained components' time series are sufficient to explain 50 percent of variance across the nuisance mask (CSF, WM, combined, or temporal). The 5 remaining components are dropped from consideration. The head-motion estimates calculated in the correction step were also placed within the corresponding confounds file. The confound time series derived from head motion estimates and global signals were expanded with the inclusion of temporal derivatives and quadratic terms for each (Satterthwaite et al.,

2013). Frames that exceeded a threshold of 0.5 mm FD or 1.5 standardised DVARS were annotated as motion outliers. All resamplings can be performed with a single interpolation step by composing all the pertinent transformations (i.e., head-motion transform matrices, susceptibility distortion correction when available, and co-registrations to anatomical and output spaces). Gridded (volumetric) resamplings were performed using antsApplyTransforms (ANTs), configured with Lanczos interpolation to minimize the smoothing effects of other kernels (Lanczos, 1964). Non-gridded (surface) resamplings were performed using mri_vol2surf (FreeSurfer).

Many internal operations of fMRIPrep use Nilearn 0.6.2 (Abraham et al., 2014), RRID:SCR_001362, mostly within the functional processing workflow. For more details of the pipeline, see the section corresponding to workflows in fMRIPrep's documentation.

2. Gradient 1 (principal gradient) validation

To confirm that our second gradient (numbered to conform with the conventions as G1) was aligned to the typical sensorimotor-to-associative axis, we performed correlations between the average gradient score of each brain region and the well-known principal gradient reported by Margulies et al., 2016 (Spearman $\rho = 0.72$; $p < 0.01$; Supplementary Figure 1a) as well as with a network hierarchy organisation (Spearman $\rho = 0.77$; $p < 0.01$; Supplementary Figure 1b).

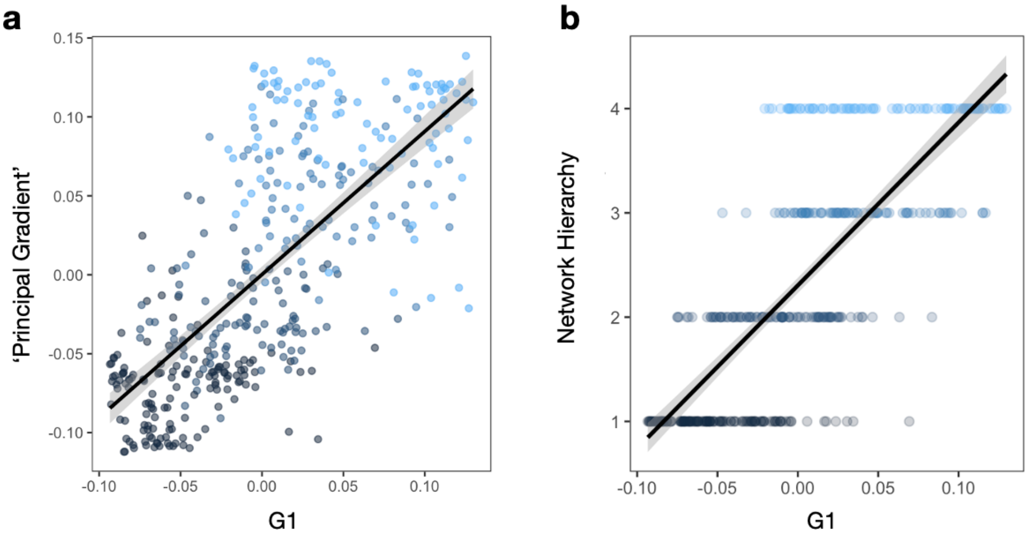

Supplementary Figure 1. a) Correlation of G1 captured in our cohort with Margulies' Principal Gradient. b) Correlation of G1 scores assigned to networks and organised into proposed network hierarchy (1 = Sensory Networks, 2 = Attention networks, 3 = Frontoparietal and Limbic Networks, 4 = Default mode networks).

3. Alternative group-level gradient generation

We estimated gradients by averaging the functional connectivity maps across participants, with the inclusion of controls. To further verify the robustness of the group-level gradient ordering, we examined the correspondence between gradients computed without controls, to confirm that this did not change the order of variance explained by the first three gradients. As can be seen below, the sensorimotor-to-associative gradient (G1) explained less variance than the visual-to-somatomotor (G2) gradient across all groups when constructed separately (Supplementary Table 1). And aligning the gradients with and without the controls did not change the ordering of gradients (Supplementary Figures 2-3).

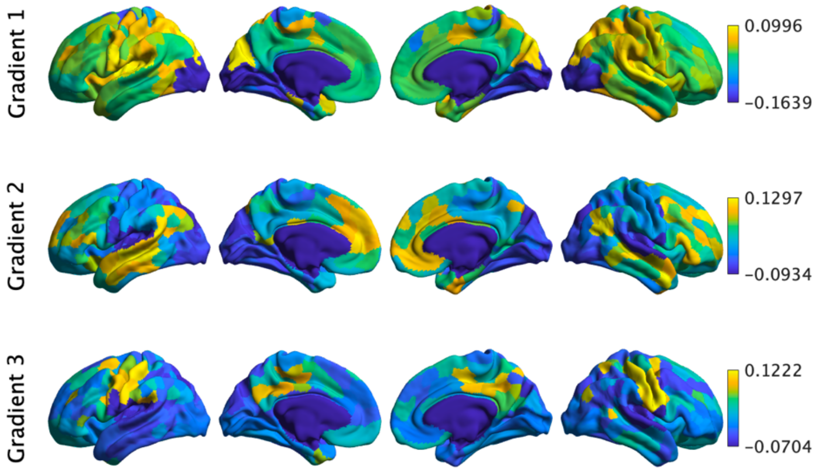

Supplementary Figure 2. Gradients generated using all participants (PD and controls).

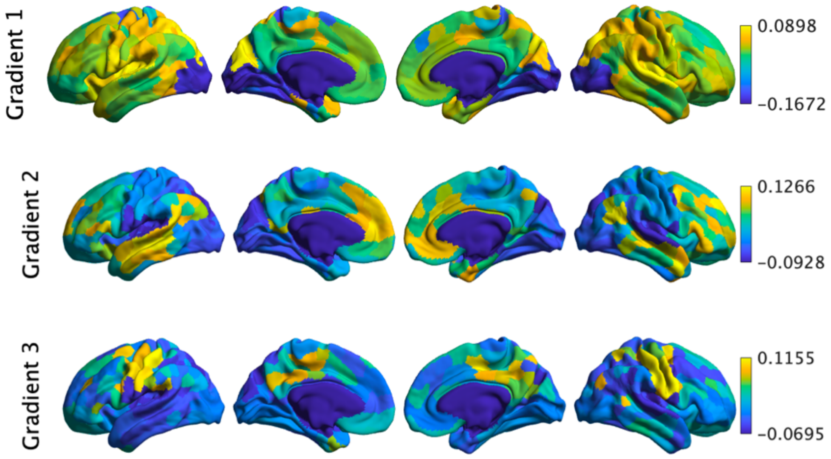

Supplementary Figure 3. Gradients generated using PD participants only.

Supplementary Table 1. First three gradients extracted for each group and the variance explained.

Cohort	Gradient	Variance explained
Controls	Visual-to-Somatomotor	15.08%
	Sensorimotor-to-Associative	13.61%
	Somatomotor-to-Insula	10.59%
PD-ON	Visual-to-Somatomotor	14.21%
	Sensorimotor-to-Associative	11.77%
	Somatomotor-to-Insula	10.26%
PD-OFF	Visual-to-Somatomotor	14.16%
	Sensorimotor-to-Associative	13.23%
	Somatomotor-to-Insula	10.33%

4. **Gradient kurtosis, skew, range and bimodality**

Assessing the gradient score distributions for each group, all distributions of the first and third gradients (G1 and G3) were slightly left-skewed (skewness > 0) and thick-tailed (kurtosis > 2). All distributions of the second gradients (G2) were slightly right-skewed (skewness < 0) and thick-tailed (kurtosis > 2). We compared the range of the gradient distribution (Supplementary Table 2) and its bimodality (Supplementary Table 3) across the groups using measures that capture changes to the distribution between gradient anchors, such that a reduction of the range or bimodality of the gradient distribution may infer the anchors moving closer to each other. Bimodality was quantified using Hartigan's dip test (Hartigan & Hartigan, 1985), a measure used previously in gradient literature (Bethlehem et al., 2020). Hartigan's dip test for unimodality quantifies the degree of multimodality in a distribution by measuring the maximum deviation between the empirical distribution function and the best-fitting unimodal distribution. The significance of the test is assessed using a null distribution, which provides p-values for rejecting the null hypothesis of unimodality.

We used mixed effects models from the 'afex' package (Singmann et al., 2020) to compare gradient range between the groups and conducted post hoc comparisons with FDR adjustments for multiple testing with the 'emmeans' package (Lenth et al., 2019). Range of G1, G2 and G3 did not significantly differ between Parkinson's disease participants ON dopamine and healthy controls, nor between Parkinson's disease participants OFF dopamine and healthy controls (Supplementary Table 2). Range of G1, G2 and G3 did not significantly differ between Parkinson's disease participants ON and OFF dopamine (Supplementary Table 2).

Supplementary Table 2. Comparison of gradient range across groups

**Gradient**	**Condition**	**Range mean (SD)**	**Comparison**
G1	PD – ON	0.197 (0.0285)	F (2, 29.088) = 2.5023, p = 0.099
	PD – OFF	0.205 (0.0314)	Pairwise:<br>PD-ON vs PD-OFF: p = 0.3811
	Controls	0.220 (0.0495)	PD-ON vs Controls: p = 0.1052<br>PD-OFF vs Controls: p = 0.3620
G2	PD – ON	0.232 (0.0374)	F (2, 33.825) =1.2767, p = 0.292
	PD – OFF	0.221 (0.0390)	Pairwise:<br>PD-ON vs PD-OFF: p = 0.2642
	Controls	0.226 (0.0419)	PD-ON vs Controls: p = 0.8679<br>PD-OFF vs Controls: p = 0.8975
G3	PD – ON	0.162 (0.0264)	F (2, 47.88) = 0.5925, p = 0.557

	PD – OFF	0.159 (0.0287)	Pairwise:<br>PD-ON vs PD-OFF: $p = 0.9404$<br>PD-ON vs Controls: $p = 0.6999$<br>PD-OFF vs Controls: $p = 0.5285$
	Controls	0.168 (0.0236)	

We did not observe significant bimodality in G1 for Controls, PD-ON and PD-OFF ($p > 0.05$; Supplementary Table 3). At the level of G2, there was evidence of bimodality across all groups, more strongly for PD-ON and PD-OFF ($p < 0.05$; Supplementary Table 3). At the level of G3, bimodality was also evident across all groups ($p < 0.05$; Supplementary Table 3).

Supplementary Table 3. Comparison of gradient bimodality across groups

Gradient	Condition	Hartigan's D statistic	Frequency distribution of gradient scores
G1	PD – ON	D = 0.005,<br>p-value = 0.070<br>$p_{FDR} = 0.105$	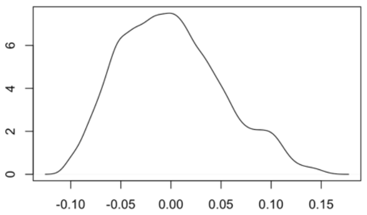
	PD – OFF	D = 0.005,<br>p-value = 0.061<br>$p_{FDR} = 0.105$	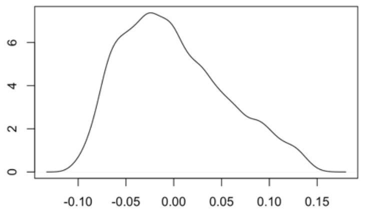
	Controls	D = 0.005,<br>p-value = 0.180<br>$p_{FDR} = 0.180$	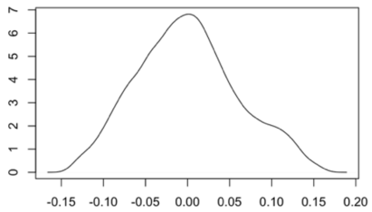
G2	PD – ON	D = 0.006,<br>p-value = 0.007<br>$p_{FDR} = 0.014$	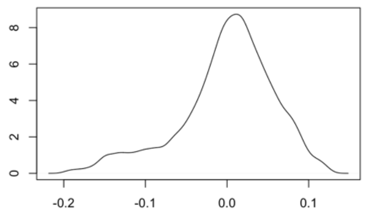
	PD – OFF	D = 0.006,<br>p-value = 0.009<br>$p_{FDR} = 0.014$	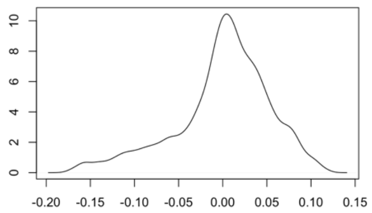

	Controls	D = 0.006,<br>p-value = 0.048<br>p_{FDR} = 0.048	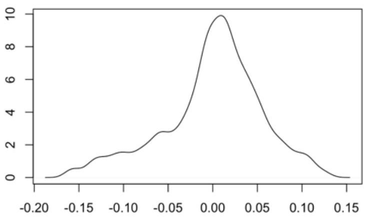
G3	PD – ON	D = 0.006,<br>p-value = 0.008<br>p_{FDR} = 0.012	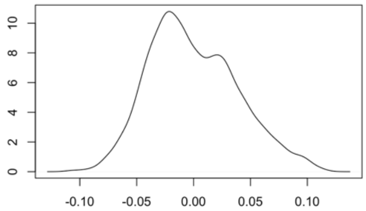
	PD – OFF	D = 0.007,<br>p-value = 0.003<br>p_{FDR} = 0.009	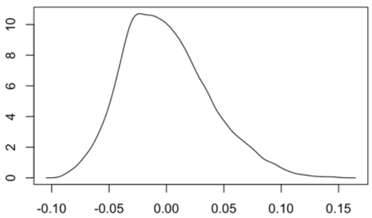
	Controls	D = 0.007,<br>p-value = 0.025<br>p_{FDR} = 0.025	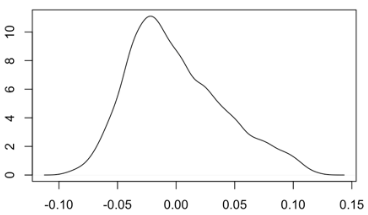

5. **List of regions with significant differences in gradient scores**

List of the significantly different regions from paired permutation testing of individuals with Parkinson's disease ON vs OFF dopamine for each gradient.

PD-ON vs PD-OFF

Supplementary Table 4. G1 regional permutation testing: PD-ON vs PD-OFF

ROI Name	MNI coordinates (X, Y, Z)			*P* value	T statistic
17Networks_LH_SomMotA_7	-4	-25	56	0.02897103	-2.2044352
17Networks_LH_SomMotA_13	-19	-24	67	0.03096903	-2.3284437
17Networks_LH_DorsAttnA_SPL_3	-23	-65	46	0.02797203	2.40429252
17Networks_LH_SalVentAttnA_ParOper_1	-55	-32	22	0.02497503	2.44692407
17Networks_LH_SalVentAttnB_OFC_1	-27	49	-14	0.01198801	2.86259441
17Networks_LH_ContA_IPS_5	-33	-46	41	0.00899101	2.90662107
17Networks_LH_ContA_PFClv_2	-42	38	22	0.00999001	2.74644011
17Networks_LH_DefaultA_pCunPCC_2	-5	-60	30	0.02997003	-2.3505689
17Networks_LH_DefaultB_Temp_5	-61	-35	-3	0.03096903	-2.3607518
17Networks_LH_DefaultB_IPL_1	-45	-58	21	0.04695305	-2.0449209
17Networks_RH_SomMotA_8	32	-34	63	0.00499501	-3.2202961
17Networks_RH_LimbicB_OFC_4	20	43	-18	0.02697303	2.18577662
17Networks_RH_ContB_PFClv_2	28	55	-14	0.00699301	2.97804401
17Networks_RH_DefaultA_pCunPCC_1	6	-52	23	0.02897103	-2.3543005
17Networks_RH_DefaultA_pCunPCC_2	5	-63	31	0.00499501	-3.0590461
17Networks_RH_DefaultA_pCunPCC_5	10	-53	35	0.00699301	-3.2710039
17Networks_RH_DefaultC_IPL_1	48	-64	22	0.01798202	-2.4644661
17Networks_RH_DefaultC_PHC_1	23	-18	-27	0.03396603	-2.4025981
17Networks_RH_TempPar_10	62	-40	22	0.02697303	2.38529846

Supplementary Table 5. G2 regional permutation testing: PD-ON vs PD-OFF

ROI Name	MNI coordinates (X, Y, Z)			*P* value	T statistic
17Networks_LH_VisCent_ExStr_5	-24	-97	-12	0.000999	-3.1604762
17Networks_LH_VisPeri_StriCal_1	-5	-88	2	0.03396603	-2.2817541
17Networks_LH_VisPeri_StriCal_2	-7	-74	9	0.01798202	-2.4062857
17Networks_LH_VisPeri_ExStrSup_1	-19	-65	7	0.00899101	-2.7887572
17Networks_LH_SomMotA_6	-9	-38	54	0.03196803	2.17533244
17Networks_LH_SomMotA_13	-19	-24	67	0.02097902	2.48072039
17Networks_LH_SomMotA_18	-19	-40	72	0.02997003	2.32164431
17Networks_LH_DorsAttnB_FEF_2	-25	-1	55	0.01798202	2.51783483
17Networks_LH_SalVentAttnA_ParOper_2	-58	-44	27	0.03696304	2.13251033

17Networks_LH_SalVentAttnA_Ins_3	-33	19	8	0.01098901	2.82441718
17Networks_LH_SalVentAttnA_FrMed_1	-7	0	41	0.02897103	2.14321976
17Networks_LH_SalVentAttnB_PFCl_2	-29	43	30	0.01998002	2.38484671
17Networks_LH_SalVentAttnB_PFCl_3	-36	32	38	0.02797203	2.15616517
17Networks_LH_ContA_Cingm_1	-3	5	29	0.02197802	2.56112702
17Networks_LH_ContB_Temp_1	-60	-36	-18	0.00599401	-2.8328996
17Networks_LH_ContB_Temp_2	-60	-49	-10	0.02697303	-2.3243874
17Networks_LH_DefaultA_pCunPCC_5	-3	-15	37	0.002997	3.79591213
17Networks_LH_DefaultA_PFCm_6	-5	34	21	0.03696304	2.34746205
17Networks_LH_TempPar_6	-59	-49	16	0.04095904	2.1233555
17Networks_RH_VisCent_Striate_1	8	-92	-2	0.02797203	-2.1602354
17Networks_RH_VisPeri_StriCal_1	9	-74	9	0.02897103	-2.4294707
17Networks_RH_DorsAttnA_ParOcc_3	36	-79	24	0.01998002	-2.3938707
17Networks_RH_DorsAttnB_PostC_7	24	-50	68	0.00899101	2.801793
17Networks_RH_DorsAttnB_PostC_8	16	-47	74	0.002997	3.3532818
17Networks_RH_SalVentAttnB_IPL_1	62	-37	37	0.01398601	2.49032406
17Networks_RH_SalVentAttnB_PFCl_3	33	45	28	0.04095904	2.05334714
17Networks_RH_LimbicA_TempPole_3	37	17	-38	0.03496504	-2.3482543
17Networks_RH_ContA_IPS_2	54	-33	51	0.01798202	2.5182628
17Networks_RH_ContA_Cingm_1	5	1	30	0.00599401	2.81565617
17Networks_RH_ContB_IPL_1	55	-45	33	0.01498502	2.46900576
17Networks_RH_ContB_IPL_3	56	-41	48	0.01498502	2.43245281
17Networks_RH_ContB_PFCld_1	39	33	38	0.002997	3.43009354
17Networks_RH_DefaultA_pCunPCC_4	4	-20	37	0.01198801	2.88374019
17Networks_RH_DefaultA_PFCm_5	17	65	16	0.00499501	3.09378408
17Networks_RH_DefaultA_PFCm_6	6	25	18	0.02197802	-2.4651399
17Networks_RH_TempPar_3	49	-20	-8	0.00699301	-2.6996717

Supplementary Table 6. G3 regional permutation testing: PD-ON vs PD-OFF

ROI Name	MNI coordinates (X, Y, Z)			*P* value	T statistic
17Networks_LH_VisCent_ExStr_1	-36	-62	-17	0.03096903	-2.3700723
17Networks_LH_VisCent_ExStr_3	-36	-81	-16	0.02597403	-2.3106408
17Networks_LH_VisCent_ExStr_11	-25	-85	21	0.04895105	-2.0540133
17Networks_LH_SomMotA_18	-19	-40	72	0.00999001	-2.8214258
17Networks_LH_DorsAttnB_PostC_7	-7	-59	63	0.01898102	-2.4966877
17Networks_LH_SalVentAttnA_ParMed_3	-6	-49	57	0.03296703	-2.1317774
17Networks_LH_ContB_IPL_1	-49	-60	47	0.00499501	-3.1691892
17Networks_LH_ContB_IPL_2	-53	-50	45	0.02297702	-2.3205494
17Networks_LH_DefaultA_pCunPCC_7	-7	-51	43	0.01198801	-2.7681506
17Networks_LH_DefaultA_PFCm_1	-5	55	-10	0.04095904	2.13886967

17Networks_LH_DefaultC_PHC_2	-30	-33	-18	0.01198801	2.61725737
17Networks_LH_TempPar_4	-52	-43	5	0.02997003	2.22766049
17Networks_RH_VisCent_ExStr_1	36	-53	-17	0.04395604	-2.0486638
17Networks_RH_SomMotA_12	29	-11	65	0.04595405	2.11170801
17Networks_RH_SomMotA_13	9	-40	68	0.01598402	-2.6862899
17Networks_RH_DorsAttnB_PostC_8	16	-47	74	0.00999001	-2.6616972
17Networks_RH_DorsAttnB_FEF_3	25	-3	64	0.03396603	2.14872301
17Networks_RH_SalVentAttnA_FrMed_1	7	2	43	0.04495505	-2.1510033
17Networks_RH_SalVentAttnA_ParMed_1	11	-17	41	0.00799201	3.01382771
17Networks_RH_SalVentAttnB_IPL_1	62	-37	37	0.01098901	-2.6701787
17Networks_RH_SalVentAttnB_Ins_1	34	21	-8	0.01398601	3.01751071
17Networks_RH_LimbicB_OFC_2	23	22	-21	0.01398601	2.64464963
17Networks_RH_LimbicB_OFC_4	20	43	-18	0.04095904	2.02985783
17Networks_RH_LimbicB_OFC_5	5	22	-21	0.002997	3.50001829
17Networks_RH_ContA_PFCl_4	49	8	25	0.02897103	2.22728965
17Networks_RH_DefaultA_PFCm_1	5	41	-11	0.03696304	2.148859
17Networks_RH_DefaultA_PFCm_3	7	42	4	0.02597403	2.25985138
17Networks_RH_DefaultB_PFCv_1	35	23	-18	0.003996	3.72673755

6. Subject level gradients in three-dimensional space

Subject level maps of three-dimensional gradient organisation.

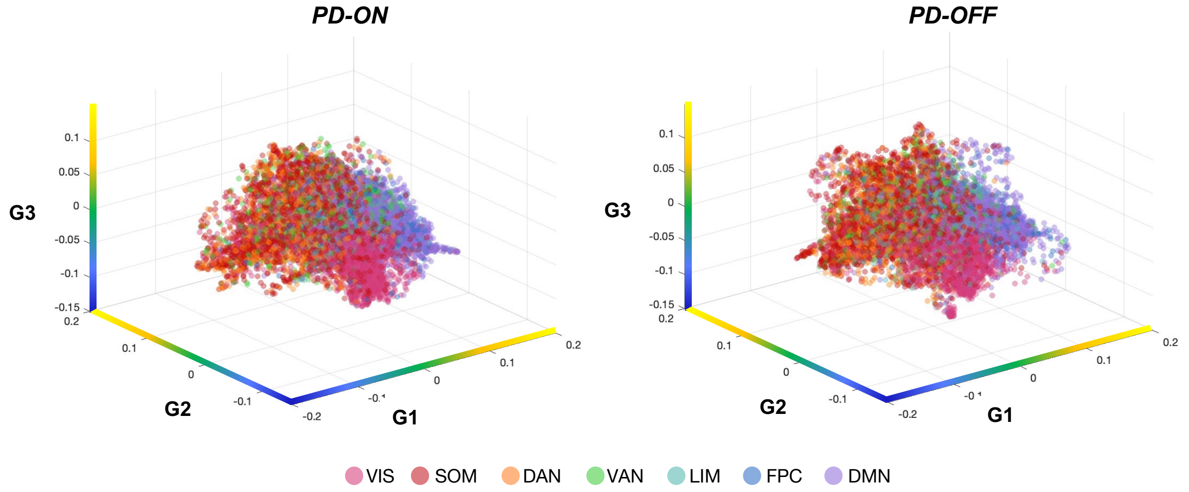

Supplementary Figure 4. Subject level functional gradients in three-dimensional space. The first three gradients projected into a three-dimensional gradient space for all subjects in the ON dopamine and OFF dopamine states. Regional points coloured by its functional network classification. Functional networks: DAN = dorsal attention; DMN = default mode network; FPC = frontoparietal control; LIM = limbic; SOM = somatomotor; VAN = ventral attention; VIS = visual.

7. Selection of explanatory variables in dispersion models

We conducted a backward elimination of fixed effects in the linear mixed models predicting dispersion to test for the selection of explanatory variables. This analysis confirmed that controlling for age and disease severity as additional regressors in the model did not qualitatively change the results.

Supplementary Table 7. Backward elimination of fixed effects in the linear mixed models predicting dispersion.

Model 1: VIS-DAN

Predictors	Model selection step		
	1	2	3
Condition (dopamine state)	-0.199*	-0.199*	-0.185*
DDE	0.200	0.191	0.134
**Condition × DDE**	-0.286**	-0.287**	-0.267**
UPDRS III	0.101	0.050	-
Age	-0.125	-	-
AIC	114.285	112.662	135.517
Δ AIC	1.623	0	22.856
BIC	128.186	124.825	147.225
Δ BIC	3.361	0	22.400

Model 2: VIS-VAN

Predictors	Model selection step		
	1	2	3
Condition (dopamine state)	-0.235*	-0.235*	-0.194*
DDE	0.288	0.274	0.176
**Condition × DDE**	-0.203*	-0.204*	-0.192*
UPDRS III	-0.069	-0.155	-
Age	-0.207	-	-
AIC	117.411	116.396	136.440
Δ AIC	1.014	0	20.044
BIC	131.312	128.560	148.148
Δ BIC	2.752	0	19.588

Values for predictors are standardised regression coefficients (ß). *p < 0.05 and **p < 0.01. Condition: ON vs. OFF dopamine medication; DDE: Dopamine Dose Equivalent; UPDRS III: Unified Parkinson's Disease Rating Scale, motor examination performed in the off-dopaminergic state; AIC: Akaike Information Criterion; BIC: Bayesian Information Criterion; Δ AIC / BIC: difference in AIC / BIC with respect to the lowest AIC / BIC value. All models included a random effect of participants on the intercept. Bolded predictor indicates interaction of interest.

When UPDRS Part III and age were added as covariates in the two linear mixed models (i.e., `VIS-DAN Dispersion ~ condition * DDE + UPDRSIII + age + (1 | subject)` and `VIS-VAN Dispersion ~ condition * DDE + UPDRSIII + age + (1 | subject)`), the results were qualitatively identical to the original models that did not include these covariates. In both dispersion models, a significant condition × DDE interaction effect and condition effect was found across all model selection steps, and no significant main effects of UPDRS III or age was uncovered.

For the Model 1, when UPDRS III was added as a covariate in the linear mixed model (i.e., `VIS-DAN dispersion ~ condition * DDE + UPDRSIII + (1 | subject)`), the results were qualitatively identical to the original model that did not include UPDRS III. There was a significant condition × DDE interaction effect (β = -0.29, F = 14.18, p = 0.001), and condition (β = -0.20, F = 6.03, p = 0.024), but no significant main effects of DDE (β = 0.19, F = 0.93, p = 0.347), UPDRS III (β = 0.05, F = 0.05, p = 0.818). When UPDRS III and age were both added as a covariate in the linear mixed model (i.e., `VIS-DAN dispersion ~ condition * DDE + UPDRSIII + age + (1 | subject)`), the results were qualitatively identical to the original model that did not include UPDRS III and age. There was a significant condition × DDE interaction effect (β = 0.27, F = 14.14, p = 0.001), and condition (β = -0.24, F = 6.01, p = 0.024) but no significant main effects of DDE (β = 0.20, F = 0.97, p = 0.338), UPDRS III (β = 0.10, F = 0.18, p = 0.673) or age (β = -0.13, F = 0.31, p = 0.585).

For the Model 2, when UPDRS III was added as a covariate in the linear mixed model (i.e., `VIS-VAN dispersion ~ condition * DDE + UPDRSIII + age + (1 | subject)`), the results were qualitatively identical to the original model that did not include UPDRS III. There was a significant condition × DDE interaction effect (β = -0.20, F = 6.36, p = 0.021), and condition (β = -0.24, F = 7.47, p = 0.013), but no significant main effects of DDE (β = 0.27, F = 1.81, p = 0.195) or UPDRS III (β = -0.16, F = 0.50, p = 0.488). When UPDRS III and age were both added as a covariate in the linear mixed model (i.e., `VIS-DAN dispersion ~ condition * DDE + UPDRSIII + age + (1 | subject)`), the results were qualitatively identical to the original model that did not include UPDRS III and age. There was a significant condition × DDE interaction effect (β = -0.20, F = 6.31, p = 0.021), and condition (β = -0.24, F = 7.43, p = 0.013) but no significant main effects of DDE (β = -0.29, F = 1.96, p = 0.179), UPDRS III (β = -0.07, F = 0.08, p = 0.776) or age (β = -0.21, F = 0.82, p = 0.378).

Together, these results point towards a greater specificity in our findings as neither age nor a measure of disease severity (UPDRS III) further explained variation in network dispersion, above and beyond the significant interaction we found between the drug state and dopamine dose.

8. Testing motion as a confounding variable in our models

As head motion is a known confounding factor in resting state studies (Van Dijk et al., 2012), we conducted analysis to assess the impact of motion (measured as the framewise displacement, indexing how much the head moves from volume to volume) on our primary network and dispersion results. First, we tested whether the magnitudes of network shifts related to changes in patients' framewise displacement between the two scans (ON minus OFF).

- G1: Difference in mean DMN value ON minus OFF dopamine did not significantly correlate with the change in framewise displacement between scans (Spearman ρ = -0.18, pFDR = 0.359).
- G2: Difference in mean VAN value ON minus OFF dopamine did not significantly correlate with the change in framewise displacement between scans (Spearman ρ = -0.06, pFDR = 0.783).
- G3: Difference in mean DMN value ON minus OFF dopamine did not significantly correlate with the change in framewise displacement between scans (Spearman ρ = -0.19, pFDR = 0.538).

To ensure our significant interactions of interest between network dispersion and dopamine dose were not confounded by motion, we included motion (measured as subject average framewise displacement in each session) as a covariate in those models. This analysis confirmed that controlling for motion did not qualitatively change our results.

Significantly different gradient dispersion across dopamine medication states - interaction effect of condition:DDE is the variable of interest:
*VIS-DAN ~ condition * DDE + motion + (1 | subject)*

- Main effect of condition: ($F(1, 24)$ = *4.370*, *p = 0.04725*)
- Main effect of DDE $F(1, 25)$ = *0.81722*, *p = 0.3747309*)
- Main effect of motion $F(1, 47)$ = *0.67316*, *p = 0.4161105)*
- **Interaction effect of condition:DDE $F(1, 25)$ = *12.96258*, *p = 0.001)***

*VIS-VAN ~ condition * DDE + motion + (1 | subject)*

- Main effect of condition: ($F(1, 24)$ = 1.11739, *p* = 0.301094)
- Main effect of DDE $F(1, 25)$ = 0.96539, *p* = 0.335518)
- Main effect of motion $F(1, 47)$ = 0.02252, *p* = 0.881360*)*
- **Interaction effect of condition:DDE $F(1, 25)$ = *6.01920*, *p = 0.022)***

9. Validating relationship between D2 receptor density and change in Gradient 2

To validate our main finding that dopamine related changes to Gradient 2 (G2) (i.e., PD-ON minus PD-OFF) was mediated by the expression of the DRD2 receptor, D2 receptor densities were also estimated using PET tracer studies (Sandiego et al., 2015; Slifstein et al., 2015; Smith et al., 2019; Zakiniaeiz et al., 2019) extracted using the *neuromaps* toolbox (Markello et al., 2022). Volumetric PET images were registered to the MNI-ICBM 152 nonlinear 2009 template and averaged across participants within each study before being parcellated to 400 cortical regions (Schaefer et al., 2018). The parcellated PET map was z-scored before correlational analysis was performed. We showed a significant correlation between the density of D2 receptors as estimated with PET maps against the change in G2 score (Spearman ρ = 0.198, p = 0.00007). This finding confirms the relationship between the change in gradient score and the D2 receptor gene expression obtained from AHBA, was also observed when estimating using PET maps.

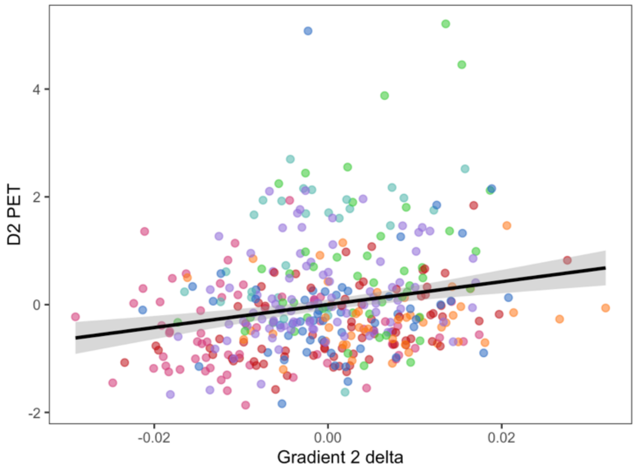

Supplementary Figure 5. *Correlating G2 changes (PD-ON minus PD-OFF; Δ) with the density of DRD2 receptor expression across the cortex (Spearman* ρ = 0.198, *p* = 0.00007*).* Functional networks: DAN = dorsal attention; DMN = default mode; FPC = frontoparietal control; LIM = limbic; SOM = somatomotor; VAN = ventral attention; VIS = visual.

10. Top 3 terms of each LDA-400 topic from the Neurosynth metanalysis

Supplementary Table 8. Spearman correlation coefficients (ρ) between the first three healthy control functional gradients and the 400 Neurosynth meta-analytic topic activation maps.

G1				G2				G3			
**Term 1**	**Term 2**	**Term 3**	**ρ**	**Term 1**	**Term 2**	**Term 3**	**ρ**	**Term 1**	**Term 2**	**Term 3**	**ρ**
'network'	'default'	'dmn'	0.42	'movements'	'movement'	'motor'	0.43	'auditory'	'sound'	'cortex'	0.64
'mpfc'	'medial'	'prefrontal'	0.35	'finger'	'tapping'	'index'	0.43	'pain'	'painful'	'chronic'	0.58
'pcc'	'cingulate'	'precuneus'	0.35	'motor'	'cortex'	'supplementary'	0.37	'speech'	'auditory'	'prosody'	0.51
'tom'	'mind'	'theory'	0.33	'motor'	'cortex'	'sensorimotor'	0.34	'music'	'musical'	'musicians'	0.49
'mental'	'mentalizing'	'states'	0.31	'hand'	'hands'	'foot'	0.33	'speech'	'sounds'	'auditory'	0.48
'social'	'cognition'	'interactions'	0.27	'force'	'motor'	'grip'	0.32	'voice'	'vocal'	'voices'	0.43
'acc'	'cingulate'	'anterior'	0.24	'stimulation'	'somatosensory'	'contralateral'	0.31	'temporal'	'sts'	'superior'	0.43
'junction'	'tpj'	'temporoparietal'	0.24	'touch'	'tactile'	'somatosensory'	0.30	'insula'	'disgust'	'insular'	0.42
'retrieval'	'memory'	'episodic'	0.24	'tms'	'stimulation'	'magnetic'	0.30	'stimulation'	'somatosensory'	'representation'	0.39
'ad'	'mci'	'disease'	0.23	'bimanual'	'sem'	'unimanual'	0.27	'stimulation'	'somatosensory'	'contralateral'	0.38
'negative'	'positive'	'valence'	0.23	'pmc'	'cord'	'sci'	0.26	'taste'	'swallowing'	'gustatory'	0.30
'amplitude'	'spontaneous'	'frequency'	0.22	'sma'	'pre'	'motor'	0.23	'touch'	'tactile'	'somatosensory'	0.27
'decision'	'making'	'choice'	0.21	'stroke'	'recovery'	'acute'	0.22	'visual'	'auditory'	'modality'	0.27
'emotional'	'emotion'	'amygdala'	0.21	'imagery'	'mental'	'imagined'	0.21	'integration'	'audiovisual'	'visual'	0.25
'schizophrenia'	'abnormalities'	'symptoms'	0.21	'tool'	'object'	'hand'	0.20	'thalamus'	'insula'	'putamen'	0.24
'reward'	'striatum'	'monetary'	0.21	'eye'	'saccade'	'saccades'	0.19	'food'	'eating'	'weight'	0.23
'dlpfc'	'prefrontal'	'cortex'	0.21	'cortex'	'primary'	'somatosensory'	0.19	'cortex'	'primary'	'somatosensory'	0.23
'judgments'	'judgment'	'judged'	0.20	'basal'	'ganglia'	'thalamus'	0.18	'autonomic'	'skin'	'arousal'	0.23
'pfc'	'prefrontal'	'cortex'	0.20	'power'	'gamma'	'hz'	0.17	'empathy'	'social'	'empathic'	0.22
'fc'	'resting'	'state'	0.19	'action'	'actions'	'observation'	0.17	'sentences'	'sentence'	'syntactic'	0.22
'cognitive'	'control'	'cognition'	0.19	'sequence'	'sequences'	'order'	0.16	'rs'	'pa'	'hyperalgesia'	0.22
'state'	'resting'	'seed'	0.19	'timing'	'rhythm'	'beat'	0.16	'ibs'	'visceral'	'rectal'	0.22
'depression'	'mdd'	'depressive'	0.19	'selection'	'pmd'	'rostral'	0.16	'language'	'hemisphere'	'linguistic'	0.22
'prefrontal'	'cortex'	'dorsolateral'	0.19	'pain'	'painful'	'chronic'	0.16	'anterior'	'insula'	'cortex'	0.21
'cortex'	'lateral'	'prefrontal'	0.19	'writing'	'drawing'	'figure'	0.15	'limbic'	'amygdala'	'paralimbic'	0.20
'error'	'errors'	'monitoring'	0.18	'stimulation'	'somatosensory'	'representation'	0.15	'comprehension'	'sentences'	'language'	0.20
'moral'	'psychopathy'	'harm'	0.18	'mi'	'withdrawal'	'sfc'	0.13	'words'	'word'	'lexical'	0.20
'memory'	'working'	'verbal'	0.18	'parietal'	'inferior'	'lobule'	0.13	'placebo'	'mg'	'blind'	0.20
'vmpfc'	'ventromedial'	'prefrontal'	0.18	'training'	'trained'	'transfer'	0.13	'ifg'	'gyrus'	'frontal'	0.20
'conflict'	'response'	'monitoring'	0.18	'mirror'	'imitation'	'observation'	0.13	'threat'	'fear'	'anxiety'	0.19
'executive'	'control'	'cognitive'	0.18	'hemisphere'	'hemispheric'	'lateralization'	0.12	'facial'	'emotion'	'expressions'	0.18

'regulation'	'emotion'	'reappraisal'	0.17	'mapping'	'scanner'	'mr'	0.12	'language'	'english'	'native'	0.18
'anterior'	'insula'	'cortex'	0.17	'ms'	'sclerosis'	'multiple'	0.11	'anxiety'	'trait'	'anxious'	0.18
'association'	'relationship'	'relationships'	0.17	'human'	'humans'	'animal'	0.11	'arousal'	'subjective'	'ratings'	0.17
'inhibition'	'response'	'stop'	0.17	'cerebellar'	'cerebellum'	'ii'	0.11	'amygdala'	'reactivity'	'affective'	0.17
'individuals'	'resonance'	'magnetic'	0.17	'parietal'	'ppc'	'posterior'	0.11	'striatum'	'striatal'	'ventral'	0.17
'cortex'	'prefrontal'	'orbitofrontal'	0.17	'inhibition'	'response'	'stop'	0.11	'regulation'	'emotion'	'reappraisal'	0.17
'salience'	'network'	'sn'	0.17	'sulcus'	'intraparietal'	'ips'	0.11	'passive'	'viewing'	'listening'	0.17
'events'	'future'	'past'	0.17	'task'	'performance'	'rest'	0.10	'naming'	'production'	'overt'	0.17
'age'	'years'	'group'	0.17	'ms'	'source'	'sources'	0.10	'brainstem'	'pag'	'ac'	0.17
'adults'	'older'	'age'	0.16	'individual'	'variability'	'inter'	0.10	'frequency'	'hz'	'slow'	0.17
'people'	'person'	'situation'	0.16	'task'	'recruitment'	'recruited'	0.10	'fear'	'conditioning'	'extinction'	0.16
'dacc'	'dorsal'	'cingulate'	0.16	'parietal'	'network'	'fronto'	0.09	'temporal'	'lobe'	'anterior'	0.16
'deception'	'truth'	'lying'	0.16	'reaction'	'time'	'times'	0.09	'olfactory'	'odor'	'odors'	0.16
'arousal'	'subjective'	'ratings'	0.16	'mental'	'rotation'	'visuospatial'	0.09	'thalamic'	'thalamus'	'pulvinar'	0.16
'task'	'switching'	'set'	0.16	'wm'	'memory'	'working'	0.09	'timing'	'rhythm'	'beat'	0.16
'autobiographical'	'memories'	'memory'	0.16	'prediction'	'predictive'	'predictions'	0.09	'ocd'	'disorder'	'compulsive'	0.16
'gyrus'	'ag'	'angular'	0.16	'instruction'	'instructed'	'instructions'	0.09	'verbs'	'verb'	'nouns'	0.15
'personality'	'traits'	'trait'	0.16	'stimulus'	'response'	'type'	0.09	'experience'	'subjective'	'experiences'	0.15
'evaluation'	'evaluations'	'esteem'	0.16	'cues'	'cue'	'cued'	0.09	'alcohol'	'substance'	'impulsivity'	0.15
'strategies'	'strategy'	'strategic'	0.16	'anterior'	'insula'	'cortex'	0.09	'response'	'hemodynamic'	'time'	0.15
'uncertainty'	'ambiguous'	'ambiguity'	0.16	'spatial'	'method'	'subject'	0.09	'light'	'shed'	'impact'	0.15
'frontal'	'inferior'	'gyrus'	0.16	'thalamus'	'insula'	'putamen'	0.09	'symptoms'	'severity'	'scores'	0.15
'items'	'recognition'	'item'	0.15	'executive'	'control'	'cognitive'	0.09	'video'	'clips'	'viewing'	0.15
'network'	'identified'	'core'	0.15	'long'	'term'	'short'	0.08	'risk'	'risky'	'taking'	0.15
'risk'	'genetic'	'relatives'	0.15	'abnormal'	'disorder'	'abnormalities'	0.08	'semantic'	'word'	'temporal'	0.15
'interference'	'stroop'	'control'	0.15	'music'	'musical'	'musicians'	0.08	'pars'	'opercularis'	'inferior'	0.15
'context'	'contextual'	'contexts'	0.15	'performance'	'task'	'cognitive'	0.08	'ms'	'source'	'sources'	0.15
'model'	'models'	'theory'	0.15	'trial'	'single'	'task'	0.08	'salience'	'network'	'sn'	0.15
'network'	'graph'	'local'	0.15	'manipulation'	'magnitude'	'simple'	0.08	'pictures'	'neutral'	'picture'	0.14
'load'	'task'	'high'	0.15	'attention'	'attentional'	'orienting'	0.08	'creative'	'creativity'	'thinking'	0.14
'age'	'development'	'adolescence'	0.15	'interaction'	'main'	'session'	0.08	'hallucinations'	'auditory'	'avh'	0.14
'parietal'	'network'	'fronto'	0.15	'number'	'numerical'	'numbers'	0.08	'central'	'capacity'	'nervous'	0.14
'question'	'unique'	'questions'	0.15	'auditory'	'sound'	'cortex'	0.08	'women'	'men'	'sex'	0.14
'high'	'level'	'resolution'	0.15	'task'	'performing'	'cognitive'	0.08	'motivation'	'avoidance'	'approach'	0.14
'wm'	'memory'	'working'	0.15	'target'	'targets'	'distractor'	0.08	'anticipation'	'anticipatory'	'aversive'	0.14
'systems'	'correlates'	'linked'	0.15	'practice'	'generation'	'retention'	0.08	'stress'	'cortisol'	'response'	0.14

'influence'	'cortex'	'influences'	0.15	'response'	'hemodynamic'	'time'	0.08	'adhd'	'disorder'	'attention'	0.14
'adolescents'	'adolescent'	'youth'	0.15	'significance'	'principal'	'methodology'	0.08	'ptsd'	'trauma'	'stress'	0.13
'sentences'	'sentence'	'syntactic'	0.15	'control'	'inhibitory'	'attentional'	0.08	'depression'	'mdd'	'depressive'	0.13
'trials'	'trial'	'response'	0.14	'trials'	'trial'	'response'	0.08	'awareness'	'conscious'	'consciousness'	0.13
'age'	'young'	'adults'	0.14	'increasing'	'level'	'time'	0.08	'infant'	'attachment'	'child'	0.13
'sustained'	'transient'	'onset'	0.14	'mechanisms'	'automatic'	'underlying'	0.08	'emotional'	'emotion'	'amygdala'	0.13
'process'	'tracking'	'run'	0.14	'linear'	'relationship'	'model'	0.08	'basal'	'ganglia'	'thalamus'	0.13
'predicted'	'individual'	'predict'	0.14	'pd'	'disease'	'parkinson'	0.08	'age'	'years'	'group'	0.13
'group'	'control'	'individuals'	0.14	'approach'	'method'	'techniques'	0.08	'gyrus'	'temporal'	'frontal'	0.13
'feedback'	'negative'	'performance'	0.14	'organization'	'hierarchical'	'organized'	0.08	'ofc'	'orbitofrontal'	'cortex'	0.13
'social'	'partner'	'interaction'	0.14	'speech'	'auditory'	'prosody'	0.08	'dopamine'	'da'	'receptor'	0.13
'problem'	'problems'	'arithmetic'	0.14	'magnetic'	'resonance'	'suggest'	0.08	'game'	'social'	'trust'	0.13
'empathy'	'social'	'empathic'	0.14	'important'	'play'	'suggest'	0.08	'faces'	'amygdala'	'emotional'	0.13
'linear'	'relationship'	'model'	0.14	'time'	'rt'	'reaction'	0.08	'treatment'	'baseline'	'follow'	0.13
'ifg'	'gyrus'	'frontal'	0.14	'resonance'	'magnetic'	'control'	0.08	'loss'	'gain'	'losses'	0.13
'performance'	'impaired'	'poor'	0.14	'resonance'	'magnetic'	'response'	0.08	'reward'	'striatum'	'monetary'	0.13
'smokers'	'smoking'	'nicotine'	0.14	'network'	'identified'	'core'	0.08	'adolescents'	'adolescent'	'youth'	0.13
'caudate'	'nucleus'	'accumbens'	0.14	'cortex'	'lateral'	'prefrontal'	0.08	'sleep'	'consolidation'	'deprivation'	0.13
'structures'	'pattern'	'contrast'	0.14	'load'	'task'	'high'	0.08	'matter'	'gray'	'volume'	0.13
'individual'	'variability'	'inter'	0.14	'neuronal'	'single'	'relevance'	0.07	'lsf'	'bladder'	'hsf'	0.13
'risk'	'risky'	'taking'	0.14	'mechanisms'	'underlying'	'unknown'	0.07	'epilepsy'	'lobe'	'temporal'	0.12
'learning'	'learned'	'associations'	0.14	'alexithymia'	'tas'	'ks'	0.07	'resonance'	'magnetic'	'response'	0.12
'prior'	'initial'	'time'	0.14	'memory'	'working'	'verbal'	0.07	'uncertainty'	'ambiguous'	'ambiguity'	0.12
'rule'	'rules'	'abstraction'	0.14	'cortex'	'anterior'	'cingulate'	0.07	'personality'	'traits'	'trait'	0.12
'verbal'	'fluency'	'task'	0.14	'learning'	'learned'	'associations'	0.07	'reading'	'phonological'	'dyslexia'	0.12
'performance'	'task'	'improved'	0.14	'interference'	'stroop'	'control'	0.07	'disease'	'atrophy'	'dementia'	0.12
'dmpfc'	'balance'	'dorsomedial'	0.14	'incompatible'	'compatibility'	'tmt'	0.07	'weight'	'lh'	'responsivity'	0.12
'frontoparietal'	'subregions'	'network'	0.14	'spatial'	'location'	'locations'	0.07	'caudate'	'nucleus'	'accumbens'	0.12
'performance'	'task'	'cognitive'	0.14	'common'	'suggest'	'overlap'	0.07	'subgroups'	'severe'	'mild'	0.12
'increasing'	'level'	'time'	0.14	'age'	'years'	'group'	0.07	'magnetic'	'resonance'	'suggest'	0.12
'placebo'	'mg'	'blind'	0.14	'visual'	'auditory'	'modality'	0.07	'knowledge'	'attributes'	'semantic'	0.12
'measures'	'measure'	'total'	0.14	'sustained'	'transient'	'onset'	0.07	'roi'	'voxel'	'rois'	0.12
'early'	'stage'	'stages'	0.14	'group'	'control'	'individuals'	0.07	'rate'	'heart'	'cardiovascular'	0.12
'prediction'	'predictive'	'predictions'	0.14	'internal'	'external'	'intention'	0.07	'frontal'	'inferior'	'gyrus'	0.12
'comprehension'	'sentences'	'language'	0.14	'frontal'	'inferior'	'gyrus'	0.07	'approach'	'method'	'techniques'	0.12
'spatial'	'method'	'subject'	0.13	'prefrontal'	'cortex'	'dorsolateral'	0.07	'hr'	'physiological'	'hypothalamus'	0.11

'relational'	'strength'	'strong'	0.13	'tactile'	'synchrony'	'synchronous'	0.07	'important'	'play'	'suggest'	0.11
'treatment'	'baseline'	'follow'	0.13	'frequency'	'hz'	'slow'	0.07	'bias'	'cr'	'biases'	0.11
'mapping'	'scanner'	'mr'	0.13	'magnetic'	'resonance'	'level'	0.07	'post'	'pre'	'ht'	0.11
'intelligence'	'md'	'fluid'	0.13	'delay'	'delayed'	'discounting'	0.07	'mood'	'affective'	'induction'	0.11
'monitoring'	'source'	'reality'	0.13	'insula'	'disgust'	'insular'	0.07	'evaluation'	'evaluations'	'esteem'	0.11
'independent'	'model'	'approach'	0.13	'provide'	'patterns'	'profile'	0.07	'female'	'male'	'gender'	0.11
'mechanisms'	'automatic'	'underlying'	0.13	'higher'	'level'	'lower'	0.07	'influence'	'cortex'	'influences'	0.11
'task'	'recruitment'	'recruited'	0.13	'human'	'provide'	'input'	0.07	'females'	'males'	'sex'	0.11
'mood'	'affective'	'induction'	0.13	'lesions'	'lesion'	'patient'	0.07	'bipolar'	'bd'	'disorder'	0.11
'understood'	'poorly'	'mechanisms'	0.13	'phase'	'phases'	'pws'	0.07	'measures'	'measure'	'total'	0.11
'task'	'difficulty'	'demands'	0.13	'prior'	'initial'	'time'	0.06	'common'	'suggest'	'overlap'	0.11
'delay'	'delayed'	'discounting'	0.13	'previous'	'research'	'reported'	0.06	'alexithymia'	'tas'	'ks'	0.11
'tinnitus'	'mindfulness'	'meditation'	0.13	'task'	'switching'	'set'	0.06	'matter'	'grey'	'volume'	0.11
'cortex'	'anterior'	'cingulate'	0.13	'cbf'	'asl'	'epi'	0.06	'cortex'	'anterior'	'cingulate'	0.11
'experience'	'subjective'	'experiences'	0.13	'conflict'	'response'	'monitoring'	0.06	'dacc'	'dorsal'	'cingulate'	0.11
'alcohol'	'substance'	'impulsivity'	0.13	'individuals'	'resonance'	'magnetic'	0.06	'pet'	'cerebral'	'rcbf'	0.10
'control'	'inhibitory'	'attentional'	0.13	'naming'	'production'	'overt'	0.06	'mechanisms'	'underlying'	'unknown'	0.10
'subgroups'	'severe'	'mild'	0.13	'performance'	'task'	'improved'	0.06	'white'	'matter'	'tensor'	0.10
'function'	'structure'	'magnetic'	0.13	'als'	'scores'	'students'	0.06	'allele'	'genotype'	'met'	0.10
'provide'	'patterns'	'profile'	0.13	'response'	'stimulus'	'contrast'	0.06	'increasing'	'level'	'time'	0.10
'internal'	'external'	'intention'	0.13	'ketamine'	'intervals'	'interval'	0.06	'suppression'	'voluntary'	'suppressed'	0.10
'patterns'	'properties'	'strongly'	0.13	'function'	'structure'	'magnetic'	0.06	'likelihood'	'consistency'	'estimation'	0.10
'correct'	'successful'	'incorrect'	0.13	'early'	'stage'	'stages'	0.06	'reliability'	'test'	'retest'	0.10
'significance'	'principal'	'methodology'	0.13	'independent'	'model'	'approach'	0.06	'stimulation'	'rtms'	'tdcs'	0.10
'higher'	'level'	'lower'	0.13	'cerebral'	'ataxia'	'sca'	0.06	'repetition'	'priming'	'repeated'	0.10
'expectancy'	'sensory'	'expectation'	0.13	'verbal'	'fluency'	'task'	0.06	'updating'	'cold'	'endogenous'	0.10
'reaction'	'time'	'times'	0.13	'rule'	'rules'	'abstraction'	0.06	'resonance'	'magnetic'	'control'	0.10
'gyrus'	'temporal'	'frontal'	0.12	'central'	'capacity'	'nervous'	0.06	'children'	'adults'	'age'	0.10
'abnormal'	'disorder'	'abnormalities'	0.12	'perspective'	'person'	'egocentric'	0.06	'competition'	'free'	'selection'	0.10
'race'	'cultural'	'chinese'	0.12	'task'	'difficulty'	'demands'	0.06	'response'	'stimulus'	'contrast'	0.10
'interaction'	'main'	'session'	0.12	'subgroups'	'severe'	'mild'	0.06	'route'	'recognition'	'forms'	0.10
'human'	'provide'	'input'	0.12	'speech'	'sounds'	'auditory'	0.06	'frontal'	'inferior'	'gyrus'	0.10
'striatum'	'striatal'	'ventral'	0.12	'structures'	'pattern'	'contrast'	0.06	'inhibition'	'response'	'stop'	0.10
'children'	'adults'	'age'	0.12	'roi'	'voxel'	'rois'	0.06	'magnetic'	'resonance'	'level'	0.10
'motivation'	'avoidance'	'approach'	0.12	'influence'	'cortex'	'influences'	0.06	'human'	'provide'	'input'	0.10
'thalamus'	'insula'	'putamen'	0.12	'systems'	'correlates'	'linked'	0.06	'set'	'identified'	'sets'	0.10

'medial'	'prefrontal'	'cortex'	0.12	'treatment'	'baseline'	'follow'	0.06	'association'	'relationship'	'relationships'	0.10
'detection'	'novelty'	'oddball'	0.12	'cognitive'	'control'	'cognition'	0.06	'phase'	'phases'	'pws'	0.09
'target'	'targets'	'distractor'	0.12	'detection'	'novelty'	'oddball'	0.06	'junction'	'tpj'	'temporoparietal'	0.09
'magnetic'	'resonance'	'suggest'	0.12	'predicted'	'individual'	'predict'	0.06	'detection'	'novelty'	'oddball'	0.09
'neuronal'	'single'	'relevance'	0.12	'attention'	'attentional'	'attended'	0.06	'medial'	'prefrontal'	'cortex'	0.09
'resonance'	'magnetic'	'control'	0.12	'interaction'	'ppi'	'psychophysiological'	0.06	'age'	'development'	'adolescence'	0.09
'phase'	'phases'	'pws'	0.12	'striatum'	'striatal'	'ventral'	0.06	'patterns'	'properties'	'strongly'	0.09
'previous'	'research'	'reported'	0.12	'pet'	'cerebral'	'rcbf'	0.05	'state'	'resting'	'seed'	0.09
'cues'	'cue'	'cued'	0.12	'frontal'	'inferior'	'gyrus'	0.05	'gmv'	'dystonia'	'pls'	0.09
'parietal'	'inferior'	'lobule'	0.12	'identified'	'independent'	'multiple'	0.05	'sexual'	'love'	'romantic'	0.09
'approach'	'method'	'techniques'	0.12	'expectancy'	'sensory'	'expectation'	0.05	'previous'	'research'	'reported'	0.09
'loss'	'gain'	'losses'	0.12	'error'	'errors'	'monitoring'	0.05	'stimulus'	'response'	'type'	0.09
'temporal'	'lobe'	'anterior'	0.12	'injury'	'tbi'	'traumatic'	0.05	'interaction'	'main'	'session'	0.09
'resonance'	'magnetic'	'response'	0.12	'autonomic'	'skin'	'arousal'	0.05	'interaction'	'ppi'	'psychophysiological'	0.09
'important'	'play'	'suggest'	0.12	'understood'	'poorly'	'mechanisms'	0.05	'insight'	'hum'	'mapp'	0.09
'language'	'hemisphere'	'linguistic'	0.12	'incongruent'	'congruent'	'congruency'	0.05	'dimensions'	'horizontal'	'ap'	0.09
'threat'	'fear'	'anxiety'	0.12	'cortex'	'prefrontal'	'orbitofrontal'	0.05	'sd'	'pupil'	'exception'	0.09
'knowledge'	'attributes'	'semantic'	0.12	'measures'	'measure'	'total'	0.05	'acc'	'cingulate'	'anterior'	0.09
'mechanisms'	'underlying'	'unknown'	0.12	'placebo'	'mg'	'blind'	0.05	'higher'	'level'	'lower'	0.09
'goal'	'goals'	'ma'	0.12	'network'	'graph'	'local'	0.05	'negative'	'positive'	'valence'	0.09
'stimulus'	'response'	'type'	0.12	'mhe'	'cirrhotic'	'lt'	0.05	'tle'	'surgical'	'sm'	0.09
'long'	'term'	'short'	0.12	'goal'	'goals'	'ma'	0.05	'individuals'	'resonance'	'magnetic'	0.08
'incongruent'	'congruent'	'congruency'	0.12	'dlpfc'	'prefrontal'	'cortex'	0.05	'smokers'	'smoking'	'nicotine'	0.08
'anticipation'	'anticipatory'	'aversive'	0.12	'taste'	'swallowing'	'gustatory'	0.05	'expectancy'	'sensory'	'expectation'	0.08
'roi'	'voxel'	'rois'	0.12	'size'	'small'	'large'	0.05	'classification'	'accuracy'	'machine'	0.08
'pairs'	'associative'	'associations'	0.12	'likelihood'	'consistency'	'estimation'	0.05	'implicit'	'explicit'	'task'	0.08
'psychosis'	'risk'	'schizophrenia'	0.12	'model'	'vestibular'	'dcm'	0.05	'context'	'contextual'	'contexts'	0.08
'creative'	'creativity'	'thinking'	0.12	'adults'	'older'	'age'	0.05	'dmpfc'	'balance'	'dorsomedial'	0.08
'ofc'	'orbitofrontal'	'cortex'	0.12	'performance'	'impaired'	'poor'	0.05	'users'	'cocaine'	'drug'	0.08
'symptoms'	'severity'	'scores'	0.12	'problem'	'problems'	'arithmetic'	0.05	'asd'	'autism'	'spectrum'	0.08
'users'	'cocaine'	'drug'	0.11	'fine'	'fcd'	'grained'	0.05	'reho'	'regional'	'homogeneity'	0.08
'likelihood'	'consistency'	'estimation'	0.11	'process'	'tracking'	'run'	0.05	'cortex'	'prefrontal'	'orbitofrontal'	0.08
'common'	'suggest'	'overlap'	0.11	'frontoparietal'	'subregions'	'network'	0.05	'identified'	'independent'	'multiple'	0.08
'task'	'performing'	'cognitive'	0.11	'representations'	'representation'	'cortex'	0.05	'prior'	'initial'	'time'	0.08
'frontal'	'inferior'	'gyrus'	0.11	'pfc'	'prefrontal'	'cortex'	0.05	'early'	'stage'	'stages'	0.08

'versus'	'implicated'	'correlates'	0.11	'reliability'	'test'	'retest'	0.05	'predicted'	'individual'	'predict'	0.08
'limbic'	'amygdala'	'paralimbic'	0.11	'modulation'	'modulated'	'modulate'	0.05	'group'	'control'	'individuals'	0.08
'search'	'lifg'	'frontal'	0.11	'strategies'	'strategy'	'strategic'	0.05	'hemisphere'	'hemispheric'	'lateralization'	0.08
'magnetic'	'resonance'	'level'	0.11	'matter'	'gray'	'volume'	0.05	'maps'	'space'	'template'	0.08
'game'	'social'	'trust'	0.11	'parietal'	'temporal'	'frontal'	0.05	'function'	'structure'	'magnetic'	0.08
'reasoning'	'hearing'	'deaf'	0.11	'identification'	'view'	'views'	0.05	'social'	'cognition'	'interactions'	0.08
'task'	'performance'	'rest'	0.11	'gyrus'	'temporal'	'frontal'	0.05	'rsfc'	'state'	'resting'	0.08
'instruction'	'instructed'	'instructions'	0.11	'dopamine'	'da'	'receptor'	0.05	'mapping'	'scanner'	'mr'	0.08
'task'	'relevant'	'irrelevant'	0.11	'rs'	'pa'	'hyperalgesia'	0.05	'intrinsic'	'extrinsic'	'hp'	0.08
'response'	'stimulus'	'contrast'	0.11	'question'	'unique'	'questions'	0.05	'systems'	'correlates'	'linked'	0.08
'organization'	'hierarchical'	'organized'	0.11	'maps'	'space'	'template'	0.04	'physical'	'itch'	'sc'	0.07
'set'	'identified'	'sets'	0.11	'intrinsic'	'extrinsic'	'hp'	0.04	'model'	'models'	'theory'	0.07
'number'	'numerical'	'numbers'	0.11	'white'	'matter'	'tensor'	0.04	'sensitive'	'sensitivity'	'suggest'	0.07
'recall'	'digit'	'span'	0.11	'focus'	'multi'	'bci'	0.04	'exclusion'	'ds'	'rejection'	0.07
'identified'	'independent'	'multiple'	0.11	'feedback'	'negative'	'performance'	0.04	'neuronal'	'single'	'relevance'	0.07
'central'	'capacity'	'nervous'	0.11	'high'	'level'	'resolution'	0.04	'provide'	'patterns'	'profile'	0.07
'competition'	'free'	'selection'	0.11	'recall'	'digit'	'span'	0.04	'gaba'	'ratio'	'levels'	0.07
'modulation'	'modulated'	'modulate'	0.11	'gaze'	'eye'	'eyes'	0.04	'als'	'scores'	'students'	0.07
'time'	'rt'	'reaction'	0.11	'range'	'wide'	'characteristic'	0.04	'verbal'	'fluency'	'task'	0.07
'semantic'	'word'	'temporal'	0.11	'passive'	'viewing'	'listening'	0.04	'risk'	'genetic'	'relatives'	0.07
'identification'	'view'	'views'	0.11	'space'	'distance'	'line'	0.04	'abnormal'	'disorder'	'abnormalities'	0.07
'infant'	'attachment'	'child'	0.11	'patterns'	'properties'	'strongly'	0.04	'size'	'small'	'large'	0.07
'real'	'world'	'nf'	0.11	'search'	'lifg'	'frontal'	0.04	'individual'	'variability'	'inter'	0.07
'anxiety'	'trait'	'anxious'	0.11	'awareness'	'conscious'	'consciousness'	0.04	'conflict'	'response'	'monitoring'	0.07
'trial'	'single'	'task'	0.11	'anticipation'	'anticipatory'	'aversive'	0.04	'real'	'world'	'nf'	0.07
'bpd'	'disorder'	'personality'	0.11	'insight'	'hum'	'mapp'	0.04	'mechanisms'	'automatic'	'underlying'	0.07
'matching'	'match'	'task'	0.11	'real'	'world'	'nf'	0.04	'understood'	'poorly'	'mechanisms'	0.07
'matter'	'gray'	'volume'	0.11	'users'	'cocaine'	'drug'	0.04	'versus'	'implicated'	'correlates'	0.07
'dopamine'	'da'	'receptor'	0.10	'decision'	'making'	'choice'	0.04	'correct'	'successful'	'incorrect'	0.07
'suppression'	'voluntary'	'suppressed'	0.10	'model'	'models'	'theory'	0.04	'internal'	'external'	'intention'	0.07
'pars'	'opercularis'	'inferior'	0.10	'suppression'	'voluntary'	'suppressed'	0.04	'pairs'	'associative'	'associations'	0.07
'attention'	'attentional'	'attended'	0.10	'sleep'	'consolidation'	'deprivation'	0.04	'search'	'lifg'	'frontal'	0.07
'awareness'	'conscious'	'consciousness'	0.10	'status'	'engaged'	'thought'	0.04	'relational'	'strength'	'strong'	0.07
'verbs'	'verb'	'nouns'	0.10	'set'	'identified'	'sets'	0.04	'structures'	'pattern'	'contrast'	0.07
'size'	'small'	'large'	0.10	'task'	'relevant'	'irrelevant'	0.04	'social'	'partner'	'interaction'	0.07
'range'	'wide'	'characteristic'	0.10	'route'	'recognition'	'forms'	0.04	'pathway'	'pathways'	'indirect'	0.07

'adhd'	'disorder'	'attention'	0.10	'rate'	'heart'	'cardiovascular'	0.04	'performance'	'impaired'	'poor'	0.07
'women'	'men'	'sex'	0.10	'state'	'resting'	'seed'	0.04	'humor'	'lc'	'na'	0.07
'pathway'	'pathways'	'indirect'	0.10	'stimulation'	'rtms'	'tdcs'	0.04	'outcome'	'born'	'outcomes'	0.07
'ocd'	'disorder'	'compulsive'	0.10	'physical'	'itch'	'sc'	0.04	'incongruent'	'congruent'	'congruency'	0.07
'test'	'tests'	'cognitive'	0.10	'symptoms'	'severity'	'scores'	0.04	'sz'	'vta'	'substantia'	0.07
'response'	'hemodynamic'	'time'	0.10	'tle'	'surgical'	'sm'	0.03	'schizophrenia'	'abnormalities'	'symptoms'	0.07
'status'	'engaged'	'thought'	0.10	'thalamic'	'thalamus'	'pulvinar'	0.03	'error'	'errors'	'monitoring'	0.07
'frequency'	'hz'	'slow'	0.10	'age'	'young'	'adults'	0.03	'illusion'	'illusory'	'contour'	0.06
'sensitive'	'sensitivity'	'suggest'	0.10	'speed'	'separation'	'mph'	0.03	'pfc'	'prefrontal'	'cortex'	0.06
'post'	'pre'	'ht'	0.10	'brainstem'	'pag'	'ac'	0.03	'race'	'cultural'	'chinese'	0.06
'female'	'male'	'gender'	0.10	'gmv'	'dystonia'	'pls'	0.03	'motor'	'cortex'	'sensorimotor'	0.06
'manipulation'	'magnitude'	'simple'	0.10	'pars'	'opercularis'	'inferior'	0.03	'sustained'	'transient'	'onset'	0.06
'pain'	'painful'	'chronic'	0.10	'medial'	'prefrontal'	'cortex'	0.03	'organization'	'hierarchical'	'organized'	0.06
'autonomic'	'skin'	'arousal'	0.10	'outcome'	'born'	'outcomes'	0.03	'action'	'actions'	'observation'	0.06
'insula'	'disgust'	'insular'	0.10	'context'	'contextual'	'contexts'	0.03	'force'	'motor'	'grip'	0.06
'rate'	'heart'	'cardiovascular'	0.10	'relational'	'strength'	'strong'	0.03	'feedback'	'negative'	'performance'	0.06
'attention'	'attentional'	'orienting'	0.10	'effort'	'fnc'	'reporting'	0.03	'independent'	'model'	'approach'	0.06
'interaction'	'ppi'	'psychophysiological'	0.10	'presented'	'visual'	'presentation'	0.03	'reasoning'	'hearing'	'deaf'	0.06
'light'	'shed'	'impact'	0.09	'syndrome'	'ts'	'japanese'	0.03	'faces'	'face'	'facial'	0.06
'lesions'	'lesion'	'patient'	0.09	'reference'	'frame'	'relations'	0.03	'spatial'	'method'	'subject'	0.06
'fear'	'conditioning'	'extinction'	0.09	'experience'	'subjective'	'experiences'	0.03	'network'	'graph'	'local'	0.06
'matter'	'grey'	'volume'	0.09	'social'	'partner'	'interaction'	0.03	'reading'	'letter'	'chinese'	0.06
'brainstem'	'pag'	'ac'	0.09	'lsf'	'bladder'	'hsf'	0.03	'high'	'level'	'resolution'	0.06
'effort'	'fnc'	'reporting'	0.09	'post'	'pre'	'ht'	0.03	'abstract'	'concrete'	'concepts'	0.06
'types'	'type'	'similarity'	0.09	'classification'	'accuracy'	'machine'	0.03	'representations'	'representation'	'cortex'	0.06
'reliability'	'test'	'retest'	0.09	'intervention'	'therapy'	'cbt'	0.03	'question'	'unique'	'questions'	0.06
'sequence'	'sequences'	'order'	0.09	'adolescents'	'adolescent'	'youth'	0.03	'face'	'faces'	'fusiform'	0.05
'passive'	'viewing'	'listening'	0.09	'empathy'	'social'	'empathic'	0.03	'lesions'	'lesion'	'patient'	0.05
'classification'	'accuracy'	'machine'	0.09	'risk'	'genetic'	'relatives'	0.03	'perception'	'perceived'	'subliminal'	0.05
'food'	'eating'	'weight'	0.09	'ibs'	'visceral'	'rectal'	0.03	'identification'	'view'	'views'	0.05
'abstract'	'concrete'	'concepts'	0.09	'acupuncture'	'stimulation'	'sa'	0.03	'range'	'wide'	'characteristic'	0.05
'allele'	'genotype'	'met'	0.09	'alcohol'	'substance'	'impulsivity'	0.03	'trial'	'single'	'task'	0.05
'stress'	'cortisol'	'response'	0.09	'monitoring'	'source'	'reality'	0.03	'control'	'inhibitory'	'attentional'	0.05
'parietal'	'temporal'	'frontal'	0.09	'association'	'relationship'	'relationships'	0.03	'ketamine'	'intervals'	'interval'	0.05
'reading'	'phonological'	'dyslexia'	0.09	'caudate'	'nucleus'	'accumbens'	0.03	'hippocampal'	'hippocampus'	'memory'	0.05
'model'	'vestibular'	'dcm'	0.09	'children'	'adults'	'age'	0.03	'perceptual'	'discrimination'	'perception'	0.05

'physical'	'itch'	'sc'	0.08	'uncertainty'	'ambiguous'	'ambiguity'	0.03	'fg'	'values'	'dbs'	0.05
'pattern'	'patterns'	'multivariate'	0.08	'food'	'eating'	'weight'	0.03	'model'	'vestibular'	'dcm'	0.05
'perspective'	'person'	'egocentric'	0.08	'motivation'	'avoidance'	'approach'	0.03	'network'	'identified'	'core'	0.05
'reference'	'frame'	'relations'	0.08	'age'	'development'	'adolescence'	0.03	'effort'	'fnc'	'reporting'	0.05
'beauty'	'aesthetic'	'art'	0.08	'light'	'shed'	'impact'	0.03	'linear'	'relationship'	'model'	0.05
'stimulation'	'rtms'	'tdcs'	0.08	'integration'	'audiovisual'	'visual'	0.03	'people'	'person'	'situation'	0.05
'females'	'males'	'sex'	0.08	'limbic'	'amygdala'	'paralimbic'	0.03	'focus'	'multi'	'bci'	0.05
'bipolar'	'bd'	'disorder'	0.08	'adhd'	'disorder'	'attention'	0.03	'significance'	'principal'	'methodology'	0.05
'pet'	'cerebral'	'rcbf'	0.08	'ventral'	'dorsal'	'visual'	0.03	'acupuncture'	'stimulation'	'sa'	0.05
'fitness'	'heuristic'	'pbd'	0.08	'sensitive'	'sensitivity'	'suggest'	0.03	'incompatible'	'compatibility'	'tmt'	0.05
'selection'	'pmd'	'rostral'	0.08	'competition'	'free'	'selection'	0.03	'matching'	'match'	'task'	0.05
'encoding'	'memory'	'recognition'	0.08	'language'	'hemisphere'	'linguistic'	0.02	'types'	'type'	'similarity'	0.04
'olfactory'	'odor'	'odors'	0.08	'test'	'tests'	'cognitive'	0.02	'strategies'	'strategy'	'strategic'	0.04
'bias'	'cr'	'biases'	0.08	'intelligence'	'md'	'fluid'	0.02	'monitoring'	'source'	'reality'	0.04
'disease'	'atrophy'	'dementia'	0.08	'matter'	'grey'	'volume'	0.02	'ms'	'sclerosis'	'multiple'	0.04
'auditory'	'sound'	'cortex'	0.08	'stress'	'cortisol'	'response'	0.02	'long'	'term'	'short'	0.04
'hr'	'physiological'	'hypothalamus'	0.08	'epilepsy'	'lobe'	'temporal'	0.02	'ventral'	'dorsal'	'visual'	0.04
'training'	'trained'	'transfer'	0.08	'pathway'	'pathways'	'indirect'	0.02	'time'	'rt'	'reaction'	0.04
'language'	'english'	'native'	0.08	'creative'	'creativity'	'thinking'	0.02	'tactile'	'synchrony'	'synchronous'	0.04
'syndrome'	'ts'	'japanese'	0.08	'bias'	'cr'	'biases'	0.02	'prefrontal'	'cortex'	'dorsolateral'	0.04
'white'	'matter'	'tensor'	0.08	'ocd'	'disorder'	'compulsive'	0.02	'cerebellar'	'cerebellum'	'ii'	0.04
'basal'	'ganglia'	'thalamus'	0.08	'female'	'male'	'gender'	0.02	'body'	'bodies'	'eba'	0.04
'human'	'humans'	'animal'	0.08	'regulation'	'emotion'	'reappraisal'	0.02	'mhe'	'cirrhotic'	'lt'	0.04
'mental'	'rotation'	'visuospatial'	0.08	'types'	'type'	'similarity'	0.02	'practice'	'generation'	'retention'	0.04
'ms'	'source'	'sources'	0.08	'versus'	'implicated'	'correlates'	0.02	'adults'	'older'	'age'	0.04
'hemisphere'	'hemispheric'	'lateralization'	0.08	'correct'	'successful'	'incorrect'	0.02	'delay'	'delayed'	'discounting'	0.04
'words'	'word'	'lexical'	0.07	'perceptual'	'discrimination'	'perception'	0.02	'moral'	'psychopathy'	'harm'	0.04
'outcome'	'born'	'outcomes'	0.07	'expertise'	'experts'	'novices'	0.02	'power'	'gamma'	'hz'	0.04
'updating'	'cold'	'endogenous'	0.07	'arousal'	'subjective'	'ratings'	0.02	'task'	'recruitment'	'recruited'	0.04
'speech'	'auditory'	'prosody'	0.07	'salience'	'network'	'sn'	0.02	'decision'	'making'	'choice'	0.04
'practice'	'generation'	'retention'	0.07	'nc'	'abeta'	'amyloid'	0.02	'goal'	'goals'	'ma'	0.03
'intrinsic'	'extrinsic'	'hp'	0.07	'game'	'social'	'trust'	0.02	'gyrus'	'fusiform'	'parahippocampal'	0.03
'speech'	'sounds'	'auditory'	0.07	'reward'	'striatum'	'monetary'	0.02	'images'	'image'	'noise'	0.03
'insight'	'hum'	'mapp'	0.07	'exclusion'	'ds'	'rejection'	0.02	'gestures'	'gesture'	'communicative'	0.03
'sd'	'pupil'	'exception'	0.07	'pattern'	'patterns'	'multivariate'	0.02	'cognitive'	'control'	'cognition'	0.03
'facial'	'emotion'	'expressions'	0.07	'matching'	'match'	'task'	0.02	'nc'	'abeta'	'amyloid'	0.03

'taste'	'swallowing'	'gustatory'	0.07	'dacc'	'dorsal'	'cingulate'	0.02	'recall'	'digit'	'span'	0.03
'representations'	'representation'	'cortex'	0.07	'anxiety'	'trait'	'anxious'	0.02	'syndrome'	'ts'	'japanese'	0.03
'asd'	'autism'	'spectrum'	0.07	'risk'	'risky'	'taking'	0.02	'speed'	'separation'	'mph'	0.03
'reho'	'regional'	'homogeneity'	0.07	'acc'	'cingulate'	'anterior'	0.02	'intelligence'	'md'	'fluid'	0.03
'maps'	'space'	'template'	0.07	'threat'	'fear'	'anxiety'	0.02	'adaptation'	'repeated'	'code'	0.03
'route'	'recognition'	'forms'	0.07	'women'	'men'	'sex'	0.02	'psychosis'	'risk'	'schizophrenia'	0.03
'thalamic'	'thalamus'	'pulvinar'	0.07	'schizophrenia'	'abnormalities'	'symptoms'	0.02	'cbf'	'asl'	'epi'	0.03
'adaptation'	'repeated'	'code'	0.07	'ec'	'sp'	'eo'	0.01	'cerebral'	'ataxia'	'sca'	0.03
'parietal'	'ppc'	'posterior'	0.06	'temporal'	'lobe'	'anterior'	0.01	'selective'	'texture'	'selectivity'	0.03
'sz'	'vta'	'substantia'	0.06	'psychosis'	'risk'	'schizophrenia'	0.01	'modulation'	'modulated'	'modulate'	0.03
'visual'	'auditory'	'modality'	0.06	'fear'	'conditioning'	'extinction'	0.01	'sma'	'pre'	'motor'	0.03
'epilepsy'	'lobe'	'temporal'	0.06	'fitness'	'heuristic'	'pbd'	0.01	'task'	'performing'	'cognitive'	0.03
'sma'	'pre'	'motor'	0.06	'deception'	'truth'	'lying'	0.01	'task'	'difficulty'	'demands'	0.03
'weight'	'lh'	'responsivity'	0.06	'verbs'	'verb'	'nouns'	0.01	'prediction'	'predictive'	'predictions'	0.03
'sexual'	'love'	'romantic'	0.06	'hallucinations'	'auditory'	'avh'	0.01	'bpd'	'disorder'	'personality'	0.03
'tle'	'surgical'	'sm'	0.06	'rsfc'	'state'	'resting'	0.01	'category'	'categories'	'categorization'	0.03
'humor'	'lc'	'na'	0.06	'beauty'	'aesthetic'	'art'	0.01	'injury'	'tbi'	'traumatic'	0.02
'preference'	'preferences'	'nirs'	0.06	'knowledge'	'attributes'	'semantic'	0.01	'ec'	'sp'	'eo'	0.02
'dimensions'	'horizontal'	'ap'	0.06	'disease'	'atrophy'	'dementia'	0.01	'age'	'young'	'adults'	0.02
'pictures'	'neutral'	'picture'	0.06	'ptsd'	'trauma'	'stress'	0.01	'preference'	'preferences'	'nirs'	0.02
'ibs'	'visceral'	'rectal'	0.06	'allele'	'genotype'	'met'	0.01	'dlpfc'	'prefrontal'	'cortex'	0.02
'cerebellar'	'cerebellum'	'ii'	0.06	'judgments'	'judgment'	'judged'	0.01	'presented'	'visual'	'presentation'	0.02
'integration'	'audiovisual'	'visual'	0.06	'hr'	'physiological'	'hypothalamus'	0.01	'performance'	'task'	'improved'	0.02
'faces'	'amygdala'	'emotional'	0.06	'reasoning'	'hearing'	'deaf'	0.01	'familiar'	'unfamiliar'	'familiarity'	0.02
'ms'	'sclerosis'	'multiple'	0.06	'ifg'	'gyrus'	'frontal'	0.01	'status'	'engaged'	'thought'	0.02
'speed'	'separation'	'mph'	0.06	'abstract'	'concrete'	'concepts'	0.01	'performance'	'task'	'cognitive'	0.02
'presented'	'visual'	'presentation'	0.06	'mood'	'affective'	'induction'	0.01	'task'	'performance'	'rest'	0.02
'ptsd'	'trauma'	'stress'	0.06	'preference'	'preferences'	'nirs'	0.01	'fc'	'resting'	'state'	0.02
'implicit'	'explicit'	'task'	0.06	'gyrus'	'ag'	'angular'	0.01	'cortex'	'lateral'	'prefrontal'	0.02
'hallucinations'	'auditory'	'avh'	0.05	'sd'	'pupil'	'exception'	0.01	'vmpfc'	'ventromedial'	'prefrontal'	0.02
'space'	'distance'	'line'	0.05	'evaluation'	'evaluations'	'esteem'	0.01	'learning'	'learned'	'associations'	0.02
'gaba'	'ratio'	'levels'	0.05	'humor'	'lc'	'na'	0.01	'motion'	'mt'	'visual'	0.02
'amygdala'	'reactivity'	'affective'	0.05	'updating'	'cold'	'endogenous'	0.01	'test'	'tests'	'cognitive'	0.02
'mtl'	'recollection'	'memory'	0.05	'reho'	'regional'	'homogeneity'	0.01	'executive'	'control'	'cognitive'	0.02
'lsf'	'bladder'	'hsf'	0.05	'females'	'males'	'sex'	0.01	'process'	'tracking'	'run'	0.02
'als'	'scores'	'students'	0.05	'adaptation'	'repeated'	'code'	0.01	'deception'	'truth'	'lying'	0.02
'cbf'	'asl'	'epi'	0.05	'sz'	'vta'	'substantia'	0.01	'reaction'	'time'	'times'	0.02

'ketamine'	'intervals'	'interval'	0.05	'sentences'	'sentence'	'syntactic'	0.01	'encoding'	'memory'	'recognition'	0.01
'exclusion'	'ds'	'rejection'	0.05	'voice'	'vocal'	'voices'	0.00	'task'	'relevant'	'irrelevant'	0.01
'fine'	'fcd'	'grained'	0.05	'weight'	'lh'	'responsivity'	0.00	'mtl'	'recollection'	'memory'	0.01
'cortex'	'primary'	'somatosensory'	0.05	'race'	'cultural'	'chinese'	0.00	'cues'	'cue'	'cued'	0.01
'voice'	'vocal'	'voices'	0.05	'dimensions'	'horizontal'	'ap'	0.00	'human'	'humans'	'animal'	0.01
'sleep'	'consolidation'	'deprivation'	0.05	'olfactory'	'odor'	'odors'	0.00	'pattern'	'patterns'	'multivariate'	0.01
'timing'	'rhythm'	'beat'	0.05	'depression'	'mdd'	'depressive'	0.00	'trials'	'trial'	'response'	0.01
'music'	'musical'	'musicians'	0.04	'gm'	'volume'	'matter'	0.00	'pd'	'disease'	'parkinson'	0.01
'rsfc'	'state'	'resting'	0.04	'dmpfc'	'balance'	'dorsomedial'	0.00	'task'	'switching'	'set'	0.01
'focus'	'multi'	'bci'	0.04	'personality'	'traits'	'trait'	0.00	'frontoparietal'	'subregions'	'network'	0.01
'acupuncture'	'stimulation'	'sa'	0.04	'infant'	'attachment'	'child'	0.00	'parietal'	'temporal'	'frontal'	0.01
'nc'	'abeta'	'amyloid'	0.04	'implicit'	'explicit'	'task'	0.00	'training'	'trained'	'transfer'	0.01
'naming'	'production'	'overt'	0.04	'smokers'	'smoking'	'nicotine'	0.00	'human'	'agents'	'agent'	0.01
'perception'	'perceived'	'subliminal'	0.04	'exercise'	'igd'	'internet'	0.00	'fine'	'fcd'	'grained'	0.01
'incompatible'	'compatibility'	'tmt'	0.04	'surface'	'colour'	'thinning'	0.00	'judgments'	'judgment'	'judged'	0.01
'ec'	'sp'	'eo'	0.04	'sexual'	'love'	'romantic'	0.00	'attention'	'attentional'	'orienting'	0.00
'ventral'	'dorsal'	'visual'	0.04	'gaba'	'ratio'	'levels'	0.00	'interference'	'stroop'	'control'	0.00
'gm'	'volume'	'matter'	0.04	'negative'	'positive'	'valence'	0.00	'rule'	'rules'	'abstraction'	0.00
'intervention'	'therapy'	'cbt'	0.04	'reading'	'phonological'	'dyslexia'	0.00	'reference'	'frame'	'relations'	0.00
'imagery'	'mental'	'imagined'	0.03	'bipolar'	'bd'	'disorder'	0.00	'gm'	'volume'	'matter'	0.00
'hippocampal'	'hippocampus'	'memory'	0.03	'loss'	'gain'	'losses'	-0.01	'mirror'	'imitation'	'observation'	0.00
'spatial'	'location'	'locations'	0.03	'pairs'	'associative'	'associations'	-0.01	'exercise'	'igd'	'internet'	0.00
'expertise'	'experts'	'novices'	0.03	'items'	'recognition'	'item'	-0.01	'surface'	'colour'	'thinning'	0.00
'injury'	'tbi'	'traumatic'	0.03	'tinnitus'	'mindfulness'	'meditation'	-0.01	'movements'	'movement'	'motor'	0.00
'rs'	'pa'	'hyperalgesia'	0.03	'asd'	'autism'	'spectrum'	-0.01	'parietal'	'network'	'fronto'	0.00
'repetition'	'priming'	'repeated'	0.03	'words'	'word'	'lexical'	-0.01	'target'	'targets'	'distractor'	0.00
'apoe'	'epsilon'	'risk'	0.03	'language'	'english'	'native'	-0.01	'attention'	'attentional'	'attended'	0.00
'mhe'	'cirrhotic'	'lt'	0.03	'color'	'shape'	'shapes'	-0.01	'expertise'	'experts'	'novices'	0.00
'cerebral'	'ataxia'	'sca'	0.03	'comprehension'	'sentences'	'language'	-0.02	'instruction'	'instructed'	'instructions'	-0.01
'perceptual'	'discrimination'	'perception'	0.02	'junction'	'tpj'	'temporoparietal'	-0.02	'intervention'	'therapy'	'cbt'	-0.01
'writing'	'drawing'	'figure'	0.02	'perception'	'perceived'	'subliminal'	-0.02	'mi'	'withdrawal'	'sfc'	-0.01
'sulcus'	'intraparietal'	'ips'	0.02	'bpd'	'disorder'	'personality'	-0.02	'sequence'	'sequences'	'order'	-0.01
'pd'	'disease'	'parkinson'	0.02	'amygdala'	'reactivity'	'affective'	-0.02	'beauty'	'aesthetic'	'art'	-0.01
'alexithymia'	'tas'	'ks'	0.02	'ofc'	'orbitofrontal'	'cortex'	-0.02	'fitness'	'heuristic'	'pbd'	-0.01
'gmv'	'dystonia'	'pls'	0.02	'moral'	'psychopathy'	'harm'	-0.03	'cortex'	'visual'	'temporal'	-0.01
'temporal'	'sts'	'superior'	0.01	'repetition'	'priming'	'repeated'	-0.03	'items'	'recognition'	'item'	-0.01

'eye'	'saccade'	'saccades'	0.01	'encoding'	'memory'	'recognition'	-0.03	'manipulation'	'magnitude'	'simple'	-0.01
'stimulation'	'somatosensory'	'representation'	0.01	'amplitude'	'spontaneous'	'frequency'	-0.03	'selection'	'pmd'	'rostral'	-0.02
'reading'	'letter'	'chinese'	0.01	'navigation'	'virtual'	'gait'	-0.03	'load'	'task'	'high'	-0.02
'navigation'	'virtual'	'gait'	0.01	'fc'	'resting'	'state'	-0.04	'mental'	'mentalizing'	'states'	-0.02
'power'	'gamma'	'hz'	0.00	'emotional'	'emotion'	'amygdala'	-0.04	'perspective'	'person'	'egocentric'	-0.02
'exercise'	'igd'	'internet'	0.00	'apoe'	'epsilon'	'risk'	-0.04	'tinnitus'	'mindfulness'	'meditation'	-0.03
'surface'	'colour'	'thinning'	0.00	'facial'	'emotion'	'expressions'	-0.04	'problem'	'problems'	'arithmetic'	-0.03
'mirror'	'imitation'	'observation'	0.00	'images'	'image'	'noise'	-0.04	'motor'	'cortex'	'supplementary'	-0.03
'action'	'actions'	'observation'	-0.01	'selective'	'texture'	'selectivity'	-0.04	'apoe'	'epsilon'	'risk'	-0.03
'mi'	'withdrawal'	'sfc'	-0.01	'pictures'	'neutral'	'picture'	-0.05	'events'	'future'	'past'	-0.03
'motor'	'cortex'	'sensorimotor'	-0.01	'people'	'person'	'situation'	-0.05	'navigation'	'virtual'	'gait'	-0.03
'familiar'	'unfamiliar'	'familiarity'	-0.01	'semantic'	'word'	'temporal'	-0.05	'memory'	'working'	'verbal'	-0.03
'color'	'shape'	'shapes'	-0.01	'vmpfc'	'ventromedial'	'prefrontal'	-0.05	'wm'	'memory'	'working'	-0.03
'video'	'clips'	'viewing'	-0.02	'ad'	'mci'	'disease'	-0.05	'imagery'	'mental'	'imagined'	-0.03
'images'	'image'	'noise'	-0.02	'video'	'clips'	'viewing'	-0.06	'stroke'	'recovery'	'acute'	-0.04
'selective'	'texture'	'selectivity'	-0.03	'faces'	'amygdala'	'emotional'	-0.06	'blind'	'sighted'	'global'	-0.04
'motor'	'cortex'	'supplementary'	-0.03	'blind'	'sighted'	'global'	-0.06	'space'	'distance'	'line'	-0.04
'category'	'categories'	'categorization'	-0.03	'mental'	'mentalizing'	'states'	-0.06	'writing'	'drawing'	'figure'	-0.05
'stimulation'	'somatosensory'	'contralateral'	-0.04	'reading'	'letter'	'chinese'	-0.07	'parietal'	'inferior'	'lobule'	-0.05
'stroke'	'recovery'	'acute'	-0.04	'events'	'future'	'past'	-0.07	'scenes'	'scene'	'ppa'	-0.05
'gyrus'	'fusiform'	'parahippocampal'	-0.05	'temporal'	'sts'	'superior'	-0.07	'visual'	'occipital'	'early'	-0.05
'touch'	'tactile'	'somatosensory'	-0.05	'familiar'	'unfamiliar'	'familiarity'	-0.08	'amplitude'	'spontaneous'	'frequency'	-0.05
'tool'	'object'	'hand'	-0.06	'motion'	'mt'	'visual'	-0.08	'ad'	'mci'	'disease'	-0.06
'tactile'	'synchrony'	'synchronous'	-0.07	'retrieval'	'memory'	'episodic'	-0.08	'retrieval'	'memory'	'episodic'	-0.06
'force'	'motor'	'grip'	-0.07	'hippocampal'	'hippocampus'	'memory'	-0.08	'object'	'objects'	'recognition'	-0.06
'gestures'	'gesture'	'communicative'	-0.07	'mpfc'	'medial'	'prefrontal'	-0.08	'color'	'shape'	'shapes'	-0.06
'illusion'	'illusory'	'contour'	-0.08	'network'	'default'	'dmn'	-0.09	'tool'	'object'	'hand'	-0.07
'blind'	'sighted'	'global'	-0.08	'gestures'	'gesture'	'communicative'	-0.09	'mpfc'	'medial'	'prefrontal'	-0.07
'pmc'	'cord'	'sci'	-0.08	'social'	'cognition'	'interactions'	-0.09	'mental'	'rotation'	'visuospatial'	-0.08
'bimanual'	'sem'	'unimanual'	-0.09	'illusion'	'illusory'	'contour'	-0.09	'number'	'numerical'	'numbers'	-0.08
'gaze'	'eye'	'eyes'	-0.09	'cortex'	'visual'	'temporal'	-0.10	'gaze'	'eye'	'eyes'	-0.08
'cortex'	'visual'	'temporal'	-0.09	'gyrus'	'fusiform'	'parahippocampal'	-0.10	'gyrus'	'ag'	'angular'	-0.08
'movements'	'movement'	'motor'	-0.10	'tom'	'mind'	'theory'	-0.10	'tom'	'mind'	'theory'	-0.08
'fg'	'values'	'dbs'	-0.10	'category'	'categories'	'categorization'	-0.10	'autobiographical'	'memories'	'memory'	-0.08
'hand'	'hands'	'foot'	-0.11	'body'	'bodies'	'eba'	-0.11	'spatial'	'location'	'locations'	-0.09

'tms'	'stimulation'	'magnetic'	-0.11	'autobiographical'	'memories'	'memory'	-0.11	'pmc'	'cord'	'sci'	-0.09
'finger'	'tapping'	'index'	-0.11	'mtl'	'recollection'	'memory'	-0.12	'parietal'	'ppc'	'posterior'	-0.09
'body'	'bodies'	'eba'	-0.12	'fg'	'values'	'dbs'	-0.12	'bimanual'	'sem'	'unimanual'	-0.09
'face'	'faces'	'fusiform'	-0.13	'object'	'objects'	'recognition'	-0.13	'eye'	'saccade'	'saccades'	-0.11
'motion'	'mt'	'visual'	-0.13	'pcc'	'cingulate'	'precuneus'	-0.13	'hand'	'hands'	'foot'	-0.11
'faces'	'face'	'facial'	-0.14	'visual'	'occipital'	'early'	-0.14	'finger'	'tapping'	'index'	-0.11
'object'	'objects'	'recognition'	-0.14	'human'	'agents'	'agent'	-0.17	'sulcus'	'intraparietal'	'ips'	-0.11
'visual'	'occipital'	'early'	-0.19	'faces'	'face'	'facial'	-0.17	'tms'	'stimulation'	'magnetic'	-0.12
'human'	'agents'	'agent'	-0.19	'face'	'faces'	'fusiform'	-0.20	'network'	'default'	'dmn'	-0.16
'scenes'	'scene'	'ppa'	-0.28	'scenes'	'scene'	'ppa'	-0.33	'pcc'	'cingulate'	'precuneus'	-0.17

11. References

Abraham, A., Pedregosa, F., Eickenberg, M., Gervais, P., Mueller, A., Kossaifi, J., Gramfort, A., Thirion, B., & Varoquaux, G. (2014). Machine learning for neuroimaging with scikit-learn. *Frontiers in Neuroinformatics*, *8*. https://doi.org/10.3389/fninf.2014.00014

Avants, B. B., Epstein, C. L., Grossman, M., & Gee, J. C. (2008). Symmetric diffeomorphic image registration with cross-correlation: Evaluating automated labeling of elderly and neurodegenerative brain. *Medical Image Analysis*, *12*(1), 26–41. https://doi.org/10.1016/j.media.2007.06.004

Behzadi, Y., Restom, K., Liau, J., & Liu, T. T. (2007). A component based noise correction method (CompCor) for BOLD and perfusion based fMRI. *NeuroImage*, *37*(1), 90–101. https://doi.org/10.1016/j.neuroimage.2007.04.042

Bethlehem, R. A. I., Paquola, C., Seidlitz, J., Ronan, L., Bernhardt, B., Consortium, C.-C., & Tsvetanov, K. A. (2020). Dispersion of functional gradients across the adult lifespan. *NeuroImage*, *222*, 117299. https://doi.org/10.1016/j.neuroimage.2020.117299

Esteban, O., Markiewicz, C. J., Blair, R. W., Moodie, C. A., Isik, A. I., Erramuzpe, A., Kent, J. D., Goncalves, M., DuPre, E., Snyder, M., Oya, H., Ghosh, S. S., Wright, J., Durnez, J., Poldrack, R. A., & Gorgolewski, K. J. (2019). fMRIPrep: A robust preprocessing pipeline for functional MRI. *Nature Methods*, *16*(1), Article 1. https://doi.org/10.1038/s41592-018-0235-4

Evans, A. C., Janke, A. L., Collins, D. L., & Baillet, S. (2012). Brain templates and atlases. *NeuroImage*, *62*(2), 911–922. https://doi.org/10.1016/j.neuroimage.2012.01.024

Fonov, V., Evans, A., Mckinstry, R., Almli, C. R., & Collins, L. (2009). Unbiased nonlinear average age-appropriate brain templates from birth to adulthood. *Neuroimage*, *47*. https://doi.org/10.1016/S1053-8119(09)70884-5

Greve, D. N., & Fischl, B. (2009). Accurate and robust brain image alignment using boundary-based registration. *NeuroImage*, *48*(1), 63–72. https://doi.org/10.1016/j.neuroimage.2009.06.060

Hartigan, J. A., & Hartigan, P. M. (1985). The Dip Test of Unimodality. *The Annals of Statistics*, *13*(1), 70–84. https://doi.org/10.1214/aos/1176346577

Jenkinson, M., Bannister, P., Brady, M., & Smith, S. (2002). Improved Optimization for the Robust and Accurate Linear Registration and Motion Correction of Brain Images. *NeuroImage*, *17*(2), 825–841. https://doi.org/10.1006/nimg.2002.1132

Jenkinson, M., & Smith, S. (2001). A global optimisation method for robust affine registration of brain images. *Medical Image Analysis*, *5*(2), 143–156. https://doi.org/10.1016/S1361-8415(01)00036-6

Lanczos, C. (1964). Evaluation of Noisy Data. *Journal of the Society for Industrial and Applied Mathematics Series B Numerical Analysis*, *1*(1), 76–85. https://doi.org/10.1137/0701007

Lenth, R., Singmann, H., Love, J., Buerkener, P., & Herve, M. (2019). *Emmeans: Estimated Marginal Means, Aka Least-Squares Means.* https://cran.r-project.org/web/packages/emmeans/emmeans.pdf

Markello, R. D., Hansen, J. Y., Liu, Z.-Q., Bazinet, V., Shafiei, G., Suárez, L. E., Blostein, N., Seidlitz, J., Baillet, S., Satterthwaite, T. D., Chakravarty, M. M., Raznahan, A., & Misic, B. (2022). neuromaps: Structural and functional interpretation of brain maps. *Nature Methods*, 1–8. https://doi.org/10.1038/s41592-022-01625-w

Power, J. D., Mitra, A., Laumann, T. O., Snyder, A. Z., Schlaggar, B. L., & Petersen, S. E. (2014). Methods to detect, characterize, and remove motion artifact in resting state fMRI. *NeuroImage*, *84*, 10.1016/j.neuroimage.2013.08.048. https://doi.org/10.1016/j.neuroimage.2013.08.048

Pruim, R. H. R., Mennes, M., van Rooij, D., Llera, A., Buitelaar, J. K., & Beckmann, C. F. (2015). ICA-AROMA: A robust ICA-based strategy for removing motion artifacts from fMRI data. *NeuroImage*, *112*, 267–277. https://doi.org/10.1016/j.neuroimage.2015.02.064

Sandiego, C. M., Gallezot, J.-D., Lim, K., Ropchan, J., Lin, S., Gao, H., Morris, E. D., & Cosgrove, K. P. (2015). Reference Region Modeling Approaches for Amphetamine Challenge Studies with [11C]FLB 457 and PET. *Journal of Cerebral Blood Flow & Metabolism*, *35*(4), 623–629. https://doi.org/10.1038/jcbfm.2014.237

Satterthwaite, T. D., Elliott, M. A., Gerraty, R. T., Ruparel, K., Loughead, J., Calkins, M. E., Eickhoff, S. B., Hakonarson, H., Gur, R. C., Gur, R. E., & Wolf, D. H. (2013). An improved framework for confound regression and filtering for control of motion artifact in the preprocessing of resting-state functional connectivity data. *NeuroImage*, *64*, 240–256. https://doi.org/10.1016/j.neuroimage.2012.08.052

Schaefer, A., Kong, R., Gordon, E. M., Laumann, T. O., Zuo, X.-N., Holmes, A. J., Eickhoff, S. B., & Yeo, B. T. T. (2018). Local-Global Parcellation of the Human Cerebral Cortex from Intrinsic Functional Connectivity MRI. *Cerebral Cortex*, *28*(9), 3095–3114. https://doi.org/10.1093/cercor/bhx179

Singmann, H., Bolker, B., Westfall, J., Aust, F., Ben-Shachar, M., Højsgaard, S., Fox, J., Mertens, U., Love, J., Lenth, R., & Christensen, R. H. B. (2020). *Package 'afex.'* https://github.com/singmann/afex

Slifstein, M., van de Giessen, E., Van Snellenberg, J., Thompson, J. L., Narendran, R., Gil, R., Hackett, E., Girgis, R., Ojeil, N., Moore, H., D'Souza, D., Malison, R. T., Huang, Y., Lim, K., Nabulsi, N., Carson, R. E., Lieberman, J. A., & Abi-Dargham, A. (2015). Deficits in Prefrontal Cortical and Extrastriatal Dopamine Release in Schizophrenia: A Positron Emission Tomographic Functional Magnetic Resonance Imaging Study. *JAMA Psychiatry*, *72*(4), 316–324. https://doi.org/10.1001/jamapsychiatry.2014.2414

Smith, C. T., Crawford, J. L., Dang, L. C., Seaman, K. L., San Juan, M. D., Vijay, A., Katz, D. T., Matuskey, D., Cowan, R. L., Morris, E. D., Zald, D. H., & Samanez-Larkin, G. R. (2019). Partial-volume correction increases estimated dopamine D2-like receptor binding potential and reduces adult age differences. *Journal of Cerebral Blood Flow & Metabolism*, *39*(5), 822–833. https://doi.org/10.1177/0271678X17737693

Tustison, N. J., Avants, B. B., Cook, P. A., Zheng, Y., Egan, A., Yushkevich, P. A., & Gee, J. C. (2010). N4ITK: Improved N3 bias correction. *IEEE Transactions on Medical Imaging*, *29*(6), 1310–1320. https://doi.org/10.1109/TMI.2010.2046908

Van Dijk, K. R. A., Sabuncu, M. R., & Buckner, R. L. (2012). The influence of head motion on intrinsic functional connectivity MRI. *NeuroImage*, *59*(1), 431–438. https://doi.org/10.1016/j.neuroimage.2011.07.044

Zakiniaeiz, Y., Hillmer, A. T., Matuskey, D., Nabulsi, N., Ropchan, J., Mazure, C. M., Picciotto, M. R., Huang, Y., McKee, S. A., Morris, E. D., & Cosgrove, K. P. (2019). Sex differences in amphetamine-induced dopamine release in the dorsolateral prefrontal cortex of tobacco smokers. *Neuropsychopharmacology*, *44*(13), 2205–2211. https://doi.org/10.1038/s41386-019-0456-y

Zhang, Y., Brady, M., & Smith, S. (2001). Segmentation of brain MR images through a hidden Markov random field model and the expectation-maximization algorithm. *IEEE Transactions on Medical Imaging*, *20*(1), 45–57. https://doi.org/10.1109/42.906424
